# Quantitative phosphoproteomics of protein kinase SnRK1 regulated protein phosphorylation in Arabidopsis under submergence

**DOI:** 10.1093/jxb/erw107

**Published:** 2016-03-29

**Authors:** Hsing-Yi Cho, Tuan-Nan Wen, Ying-Tsui Wang, Ming-Che Shih

**Affiliations:** ^1^Molecular and Biological Agricultural Sciences Program, Taiwan International Graduate Program, National Chung-Hsing University, Academia Sinica, Taiwan; ^2^Agricultural Biotechnology Research Center, Academia Sinica, Taiwan; ^3^Institute of Plant and Microbial Biology, Academia Sinica, Taiwan; ^4^Graduate Institute of Biotechnology, National Chung-Hsing University, Taiwan; ^5^Biotechnology Center, National Chung-Hsing University, Taichung, Taiwan

**Keywords:** Energy starvation, phosphoproteomics, SnRK1, submergence.

## Abstract

SNF1 RELATED PROTEIN KINASE 1 (SnRK1) is proposed to be a central integrator of the plant stress and energy starvation signalling pathways. We observed that the Arabidopsis SnRK1.1 dominant negative mutant (*SnRK1.1* ^*K48M*^) had lower tolerance to submergence than the wild type, suggesting that SnRK1.1-dependent phosphorylation of target proteins is important in signalling pathways triggered by submergence. We conducted quantitative phosphoproteomics and found that the phosphorylation levels of 57 proteins increased and the levels of 27 proteins decreased in Col-0 within 0.5–3h of submergence. Among the 57 proteins with increased phosphorylation in Col-0, 38 did not show increased phosphorylation levels in *SnRK1.1* ^*K48M*^ under submergence. These proteins are involved mainly in sugar and protein synthesis. In particular, the phosphorylation of MPK6, which is involved in regulating ROS responses under abiotic stresses, was disrupted in the *SnRK1.1* ^*K48M*^ mutant. In addition, PTP1, a negative regulator of MPK6 activity that directly dephosphorylates MPK6, was also regulated by SnRK1.1. We also showed that energy conservation was disrupted in *SnRK1.1* ^*K48M*^, *mpk6*, and *PTP1* ^*S7AS8A*^ under submergence. These results reveal insights into the function of SnRK1 and the downstream signalling factors related to submergence.

## Highlight

To illustrate the hypoxia signal mediated by SnRK1.1, phosphoproteomics was used to identify not only the new SnRK1.1 putative targets related to submergence, but also to gain an insight into early hypoxia signalling events.

## Introduction

Owing to the limited availability of oxygen in water, plants cannot obtain sufficient energy from the oxidative phosphorylation of mitochondria to maintain their necessary functions, such as growth and defence responses to environmental changes, when submerged. Consequently, in order to avert an energy crisis during submergence plants have to adjust their metabolism. To maintain ATP production, plants switch to anaerobic fermentation. In addition, under hypoxia, plants also accumulate alanine and GABA through the GABA shunt. Alanine and GABA are used to store carbon and nitrogen which are easily lost during oxygen deprivation (Miyashita *et al.*, 2007). To control energy consumption, dynamic polysome loading of mRNAs is reduced by 50% and only specific stress response genes, such as hypoxia core response genes, are selected to the polysome (Mustroph *et al.*, 2009). However, how plants sense an energy crisis and modulate their responses under submergence remains unclear.

Two types of kinases are known to play important roles in oxygen deprivation. The activities of MPK3 and 6 are induced by the ROS released from mitochondria in the early stages of hypoxia and overexpression of MPK6 enhances anoxia tolerance in Arabidopsis (Chang *et al.*, 2012). Furthermore, SNF1 RELATED PROTEIN KINASE 1 (SnRK1) is known to trigger a vast array of transcriptional and metabolic reprogramming in response to declining energy levels (Crozet *et al.*, 2014). SnRK1 is an evolutionarily conserved energy-sensing protein kinase which is known as AMP-DEPENDENT PROTEIN KINASE 1 (AMPK) in mammals and SUCROSE NON-FERMENTING 1 (SNF1) in yeast (Polge and Thomas, 2007). In mammals, AMPK is activated by an increase in the cellular AMP/ATP ratio to enhance glucose uptake. In yeast, SNF1 plays a key role in shifting fermentation to oxidative phosphorylation in order to generate ATP efficiently under glucose starvation (Hardie *et al.*, 2011). In plants, SnRK1 is regulated by AMP (Sugden *et al.*, 1999), Ca^2+^ signalling (Lu *et al.*, 2007), GEMINIVIRUS RAP INTERACTING KINASE 1/2 (GRIK1/2) (Shen *et al.*, 2009), glucose-6-phosphate (Nunes *et al.*, 2013), and trehalose-6 phosphate (Zhang *et al.*, 2009). In Arabidopsis, two SnRK1s, SnRK1.1 (akin10) and SnRK1.2 (akin11), were proposed to be the central hub of a transcriptional network that responds to abiotic stresses and energy signalling (Baena-Gonzalez *et al.*, 2007). Two amino acids, K48 and T175 of SnRK1.1 and K49 and T176 of SnRK1.2, are essential for kinase activity. The conserved lysine residue (K48) of SnRK1.1 is required for ATP binding and the T175 is located in the activation loop of SnRK1.1 (Baena-Gonzalez *et al.*, 2007). The T175 of SnRK1.1 is phosphorylated by GRIKs (Crozet *et al.*, 2014). Phosphorylated SnRK1.1 has a stronger activity than non-phosphorylated SnRK1.1. SnRK1.1 is responsible for the major part of the activity of SnRK1 in sugar and ABA signalling (Jossier *et al.*, 2009; Rodrigues *et al.*, 2013) and miRNA-mediated energy signalling and development (Tsai and Gazzarrini, 2012; Confraria *et al.*, 2013). Under submergence, SnRK1.1 also regulates *ADH1* and *PDC1* expression, and the transgenic line that overexpresses inactive SnRK1.1 shows sensitivity to submergence (Cho *et al.*, 2012). In addition, SnRK1.1 forms complexes with β and γ proteins or with β and βγ proteins to phosphorylate the targets (Baena-Gonzalez *et al.*, 2007; Ramon *et al.*, 2013). The well-known SnRK1 targets are SUCROSE PHOSPHATE SYNTHASES (SPS), TREHALOSE PHOSPHATE SYNTHASE (TPS) (Zhang *et al.*, 2009), and FRUCTOSE-2,6 PHOSPHATE BIPHOSPHATASE (F2KP). However, the environmental conditions that trigger SnRK1 to phosphorylate these targets in plants are not clear, and the signalling mechanism of SnRK1.1 is not well elucidated.

Through AMP/ATP measurement and SnRK1 phosphorylation analysis, we found here that energy starvation occurred in the early stage (30min) of submergence in the dark. The transgenic lines, SnRK1.1^K48M^ and SnRK1.1^T175A^, that overexpress inactive forms of SnRK1.1 were sensitive to submergence and showed disrupted energy homeostasis in the late stage of submergence. We also performed iTRAQ to compare the amounts of phosphorylated peptides in Col-0 and *SnRK1.1* dominant negative mutants under submergence. The results suggest that, in the early stage of submergence, SnRK1 plays an essential role in regulating multiple cellular responses, including anaerobic metabolism, PTP1-MPK6 signalling, protein synthesis, and osmotic regulation.

## Materials and methods

### Plant materials

Transgenic lines expressing SnRK1^T175A^, SnRK1^T175D^, and SnRK1^K48M^ in Col-0 were generated by T-DNA transformation containing the 35S promoter driving the ATP binding site mutant [SnRK1^K48M^] and catalytic site mutants [SnRK1^T175D^ and SnRK1^T175A^] in the p*MDC32* vector. We analysed *SnRK1* expression and the submergence phenotype in seedlings of the T2 or T3 generations from at least two independent transgenic lines. The *cMYC-SnRK1* ^*K48M*^ overexpression lines were generated by transforming the *35S::cMYC*-*SnRK1* ^*K48M*^/*pEarleyGate 203* vector into Col-0. The T-DNA insertion lines of *mpk6* (SALK-127507) and *ptp1* (SALK-118658C) were obtained from the ABRC, Ohio State University. The PTP1^S7AS8A^ overexpression line was generated by transforming the *35S::PTP1* ^*S7AS8A*^ *-YFP*/*pEarleyGate 103* construct into *ptp1*.

All seeds were sterilized with 0.5% sodium hypochlorite for 10min and washed with sterilized water and then incubated with sterilized water at 4 °C in the dark for 3 d to achieve uniform germination. Seeds were sown on plates with 0.55% phytagel (Sigma-Aldrich) in half-strength Murashige and Skoog (MS) medium (Duchefa Biochemie) containing 0.5% sucrose at pH 5.7. The plates were transferred to a growth chamber at 22 °C with a 16h light (81 μmol m^−2^ s^−1^)/8h dark cycle and placed vertically.

### Submergence treatments

For phenotypic assays, data were collected from 36 plants per genotype in six independent experiments. Four-week-old potted plants were put into distilled water at a depth of at least 5cm from the water surface in the dark in a growth chamber under a cycle of 9h light (81 μmol m^−2^ s^−1^ at 22 °C) and 15h dark (at 18 °C). After the submergence treatment, plants were put back into a growth chamber under a 9h light (81 μmol m^−2^ s^−1^ at 22 °C) and 15h dark (at 18 °C) cycle for 4 d of recovery and then counted for damage. The percentage necrotic area of a leaf was used as the damage index. For the submergence treatment of 9-d-old seedlings, plates with plants on the surface of the medium containing half-strength MS salts and 0.5% sucrose at pH 5.7 were placed into half-strength MS liquid medium that was bubbled with 3% oxygen balanced with nitrogen for 0.5h, 1h, and 3h at room temperature in the dark. The MS liquid medium was pre-bubbled with 3% oxygen for 1h before use (the final oxygen concentration of the medium was 0.006%).

### ATP and AMP assay

Whole seedlings (fresh weight 80mg) from 9-d-old Col-0*, SnRK1* ^*T175A*^, *SnRK1* ^*T175D*^, *SnRK1* ^*K48M*^, *mpk6, ptp1*, and *PTP1* ^*S7AS8A*^ */ptp1*, treated with different times of submergence, were collected and then used to quantify ATP and AMP -with an LC mass spectrometer. The detailed conditions of LC mass spectrometry are provided in the Supplementary data at *JXB* online.

### Sample preparations for phosphoproteomics and proteomics

Sample preparations of phosphoproteomics and proteomics were performed according to Lan *et al.* (2012), and the workflow is shown in Supplementary Fig. S3 at *JXB* online. In brief, total protein was extracted from 9-d-old whole seedlings of Col-0 and *SnRK1.1* ^*K48M*^ treated with 0h, 0.5h, 1h, and 3h of submergence. The protein samples were reduced and alkylated by using dithiothreitol (DTT) and iodoacetamide (IAA). Subsequently, endoproteinase Lys-C (Wako) and trypsin (Promega) were added to digest the proteins into peptides. Then, the resulting peptide solution was acidified and desalted.

### iTRAQ labelling

For phosphoproteomics, samples of Col-0 that underwent different durations of submergence (0h, 0.5h, 1h, and 3h) were labelled 113, 114, 115, or 116, respectively, and samples from *SnRK1.1* ^*K48M-5*^ that also underwent different durations of submergence were labelled 117, 118, 119, or 121, respectively. To reduce the peptide complexity of the eight samples, after labelling each reaction mixture was aliquoted into two parts of equal volume and these aliquots were mixed into three fractions as follows: fraction 1: 113,114,115, and 116; fraction 2: 117,118,119, and 121; fraction 3: 113,114,117, and 118. The profile of each fraction in different biological repeats is listed in Supplementary Table S1 at *JXB* online.

For total proteome quantitative analysis, the desalted peptide samples from Col-0 with different treatments were labelled 113–116, and samples from *SnRK1.1* ^*K48M-5*^ with different treatments were labelled 117–121, respectively.

### Proteomics database search, quantification, and statistical analysis

Protein identification, phosphorylation modification, and quantitative analyses were analysed by the Proteome Discoverer software (ver. 1.3, Thermo Scientific). MS data were searched against the Arabidopsis protein database, TAIR10, downloaded from the Arabidopsis Information Resource Center. The data were normalized with the value of the 113-labelled sample and the median of the log_2_ ratio in each sample. The median ratio of each sample was derived from the frequency distribution histograms of log_2_ protein/phosphorylated peptide ratios of each sample.

In the phosphoproteome quantitative analysis, only unique peptides with a pRS score >50 were used for quantiﬁcation. To integrate fraction 1 and fraction 3 in each batch, the values of fraction 3 were normalized by the average value of 117/113 in fraction 2 to get the ratio of 118/113, 119/113, and 121/113. The peptides detected in two of the three biological experiments were selected for bootstrap statistical analysis (de la Fuente van Bentem *et al.*, 2008; Cleries *et al.*, 2012). The time point data that were not significantly different (*P* <0.05) are marked as “nosig”. The time point data that were not detected or detected once in three batches are marked as NA (not available).

### Recombinant protein expression and *in vitro* kinase assay

The recombinant proteins of F2KP, PENTA, eIFiso4G1, and PTP1 were purified from *E. coli*. For the *in vitro* kinase assay, Immunoprecipitated SnRK1.1 from Col-0 was incubated with recombinant protein (1–0.8 μg) in 20 μl of kinase buffer for 1h at 30 °C. The kinase reactions were performed with or without λ phosphatase (New England Biolabs) for 1h at 30 °C. All reaction products were resolved in a Phos Tag SDS PAGE (Wako) and S-tag immunoblotting. The details of recombinant protein expression and *in vitro* kinase assay are provided in the Supplementary data.

### Metabolomics extraction and quantification

The ground tissue (100mg) from 9-d-old whole seedlings of Col-0*, SnRK1.1* ^*K48M-5*^, and *SnRK1.1* ^*K48M-9*^ treated for different durations of submergence were re-suspended with 1ml 70% methanol with 12 μg ml^–1^ ribitol, and then sonicated for 30min at 4 °C. Subsequently, samples were centrifuged at the highest speed at 4 °C for 15min. The supernatant of each sample was collected and vacuum dried. The dried samples were then derivatized by *bis*(trimethylsilyl)-trifluoroacetamide (BSTFA) containing 1% trimethylchlorosilane (TMCS) and analysed using a Pegasus 4D GCxGC-TOFMS system (LECO, St Joseph, MI, USA). The detailed conditions of GCxGC-TOFMS system are provided in the Supplementary data.

### Bimolecular fluorescence complementation

For the BIFC assay, the SnRK1.1, PTP1, and MPK6 coding regions were constructed into the destination expression vectors 3130 (pSAT-35S:: cYFP-DEST) and 3136 (pSAT-35S::nYFP-DEST), respectively. The resulting 3130-target and 3136-SnRK1.1 constructs were mixed and transfected into Arabidopsis leaf protoplasts (Wu *et al.*, 2009). The protoplasts were visualized with a Zeiss LSM510 META laser scanning confocal microscope.

### Arabidopsis transient transformation

Arabidopsis transient assays were performed using 7-d-old seedlings infiltrated with *Agrobacterium* C58Ci carrying 35S::PTP1-YFP, 35S::PTP1^S7AS8A^-YFP, or 35S::PTP1^S7DS8D^-YFP in vector pEarlygate 103 according to Wu *et al.* (2014). For examination of the interaction of MPK6 and transiently expressed PTP1, PTP1^S7AS8A^-YFP, or PTP1^S7DS8D^-YFP, and different forms of PTP1 were immunoprecipitated by an anti-GFP antibody. The steps of protein immunoprecipitation were the same as those used in the *in vitro* kinase assays. The pull-down protein mixture was further examined by MPK6 and GFP immunoblotting.

### Accession numbers

The two proteomics datasets were deposited to the ProteomeXchange Consortium via the PRIDE partner repository (Vizcaino *et al.*, 2014) with the data set identifier PXD001815 for phosphoprotoemics data and PXD002353 for total proteomics data. The primer sequences used in this study, including gene accession numbers, are listed in Supplementary Table S8.

## Results

### AMP/ATP ratio increases in Arabidopsis cells under submergence

**Fig. 1.**
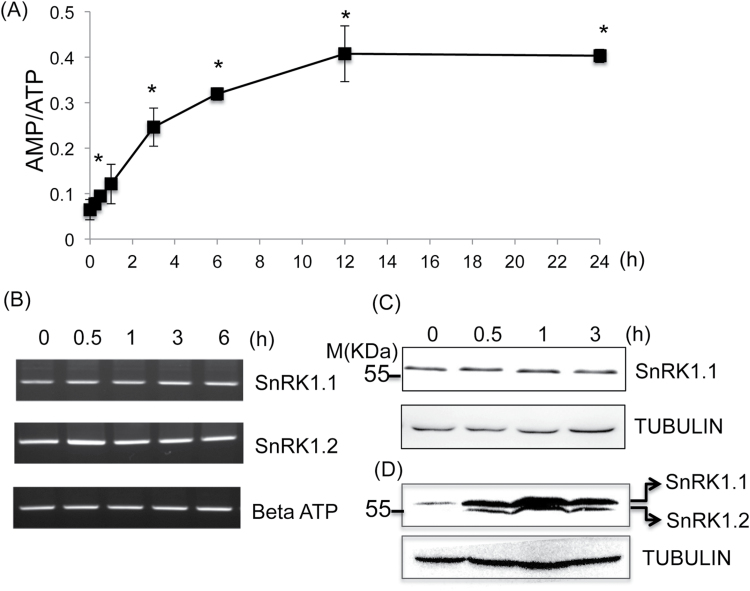
Energy starvation occurs in 9-d-old seedlings in the early stage of submergence at room temperature in the dark. (A) The change in the AMP/ATP ratio in Col-0 in the early stage of submergence. (B) The expression of *SnRK1.1* and *SnRK1.2* in Col-0 under submergence. The expression of βATP was used as an internal control. (C) Protein abundance and (D) phosphorylation of SnRK1 under submergence. According to Baena-Gonzalez *et al.* (2007) and the molecular weight of SnRK1.1 (61kDa) and SnRK1.2 (58kDa), the upper band represents the phosphorylation of SnRK1.1^T175^ and the lower band represents the phosphorylation of SnRK1.2^T175^. TUBULIN was used as an internal control. The data represent four independent biological replicates. Statistical differences between submerged and control samples were determined by Student’s *t* test. *, *P* <0.05.

To control cellular metabolism, energy sensing is usually achieved in the cell by monitoring of the AMP/ATP ratio (Hardie, 2011). To uncover the time at which energy starvation occurs under submergence, we quantified the cellular levels of ATP and AMP by LC-MS. The results showed that the AMP/ATP ratio increased within 30min of submergence, indicating the occurrence of energy starvation during submergence. Notably, after 6h of submergence, the AMP/ATP ratio reached a steady-state level (Fig. 1A). These observations suggest that plants have the ability to adjust energy production and energy consumption under submergence.

SnRK1 was proposed as a signal hub of energy starvation (Baena-Gonzalez *et al.*, 2007). Therefore, we examined whether SnRK1 is regulated during the early stage of submergence by quantifying the transcripts, protein abundance, and phosphorylation of SnRK1 (Fig. 1B–D; Fig. S1). The transcripts of SnRK1.1 and SnRK1.2 were not changed under submergence (Fig. 1B). Since SnRK1.1 is a major SnRK1 isoform (Jossier *et al.*, 2009), we generated a specific SnRK1.1 antibody to detect the SnRK1.1 protein and used the p-AMPKα (T172) antibody to examine the phosphorylation level of SnRK1 under submergence. The p-AMPKα (T172) antibody recognizes threonine phosphorylation of the two SnRK1 isoforms (T175 of SnRK1.1 and SnRK1.2) (Baena-Gonzalez *et al.*, 2007). The results showed that SnRK1.1 protein abundance was not changed under submergence, but that phosphorylation of SnRK1.1 was induced within 0.5–1h of submergence (Fig. 1C, D). Interestingly, phosphorylation of SnRK1.2 was also induced at the same stage of submergence. These results suggest that SnRK1 is involved in the early signalling response of submergence.

### SnRK1.1 is involved in maintaining energy balance under submergence

**Fig. 2.**
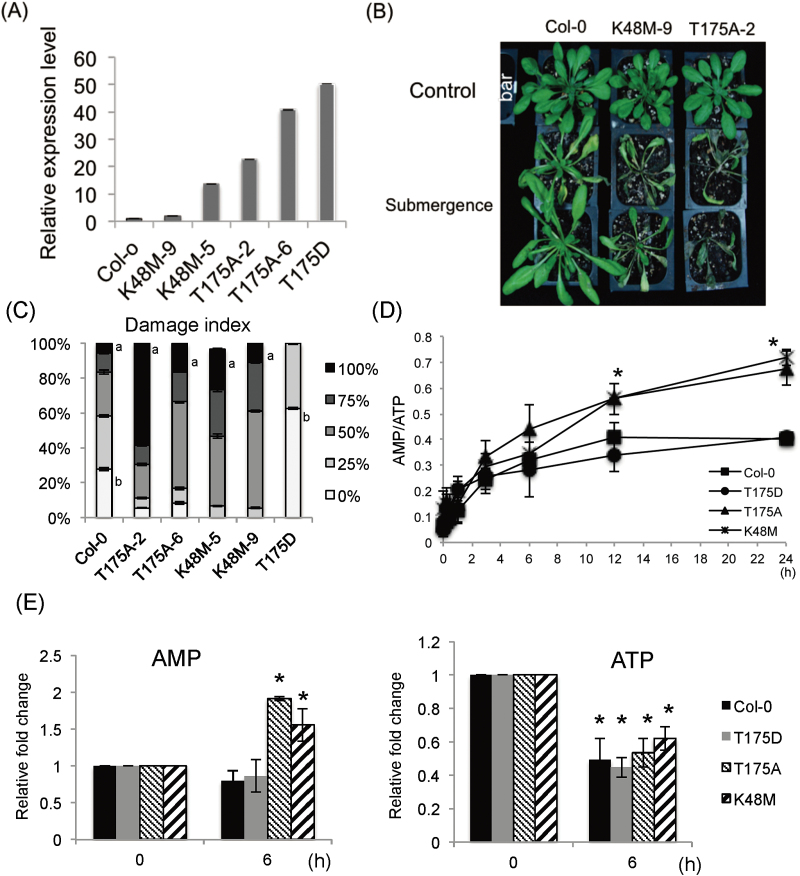
Dominant negative mutants are more sensitive to submergence than Col-0. (A) The expression of *SnRK1.1* in Col-0, *SnRK1.1* ^*K48M*^, *SnRK1.1* ^*T175A*^, and *SnRK1.1* ^*T175D*^ quantified by qRT-PCR. *TUBULIN* mRNA was the internal control. (B) The phenotype of 4-week-old Col-0 and dominant negative mutants (*SnRK1.1* ^*K48M*^, *SnRK1.1* ^*T175A*^, and *SnRK1.1* ^*T175D*^), after submergence for 60h in the dark at 22 °C and recovery for 4 d, bar =1cm. (C) After submergence for 60h and recovery for 4 d, the percentage of necrotic leaf area was used as the damage index. The data represent means ±SE from six independent biological replicates. Data from the lines with the same lowercase letters were significantly different from Col-0, which was determined by Student’s *t* test (*P* <0.05). (D) The change in the AMP/ATP ratio in 9-d-old seedlings of Col-0 and *SnRK1.1* mutants at different stages of submergence at room temperature in the dark. (E) The relative fold change of ATP and AMP in 9-d-old seedlings of Col-0 and *SnRK1.1* mutants under submergence. The data represent means ±SD from four independent biological replicates. Statistical differences between submerged and control samples were determined by Student’s *t* test. *, *P* <0.05.

Prior studies indicated a potential functional redundancy of SnRK1.1 and SnRK1.2 in hypoxia responses (Baena-Gonzalez *et al.*, 2007). Thus transgenic Arabidopsis expressing the inactive forms of SnRK1.1 (SnRK1.1^K48M^ and SnRK1.1^T175A^) were generated. We selected two SnRK1.1^K48M^, two SnRK1.1^T175A^ mutants, and one SnRK1.1^T175D^ mutant that have different transcript and protein levels of the SnRK1 transgene, for further analysis (Fig. 2A; Supplementary Fig. S2A). We first examined the submergence tolerance of these transgenic lines by subjecting 4-week-old plants to submergence for 2.5 d (60h) and recovery for 4 d. The percentage necrotic area of a leaf was used as the damage index (Fig. 2B). The results showed that the dominant negative mutants (SnRK1.1^K48M^ and SnRK1.1^T175A^) were more sensitive to submergence (Fig. 2B, C). To examine whether these physical responses were related to SnRK1.1 abundance and activity, we overexpressed the constitutive active form (SnRK1.1^T175D^) in Col-0 according to the results of (Avila *et al.*, 2012) showing that SnRK1^T175D^ has stronger activity than SnRK1.1. The transgenic plant *SnRK1.1* ^*T175D*^ with higher SnRK1.1 protein and activity had better tolerance toward submergence than Col-0 (Fig. 2C; Supplementary Fig. S2B).

We quantified the ATP and AMP levels in Col-0, *SnRK1.1* ^*K48M-5*^, *SnRK1.1* ^*T175A-2*^, and *SnRK1.1* ^*T175D*^ plants. At 0.5–3h of submergence, the AMP/ATP ratios were similar in the mutants and Col-0. After 6h of submergence, the AMP/ATP ratio in Col-0 and *SnRK1.1* ^*T175D*^ reached a steady state, but the AMP/ATP ratio in *SnRK1.1* ^*K48M-5*^ and *SnRK1.1* ^*T175A-2*^ continued to increase (Fig. 2D). These results demonstrated that inactive SnRK1.1 disrupted the ability of the cells to maintain the energy balance during the later stages of submergence. Interestingly, under submergence, the ATP level decreased in all four lines (Fig. 2E), suggesting that the energy reduction caused by oxygen deprivation was not impacted by SnRK1.1. However, the AMP accumulated to higher levels in *SnRK1.1* ^*K48M-5*^ and *SnRK1.1* ^*T175A-2*^ than in Col-0 and *SnRK1.1* ^*T175D*^ (Fig. 2E), indicating that SnRK1.1 was involved in energy conservation under submergence.

### SnRK1.1 is a central phosphorylation hub under submergence

**Fig. 3.**
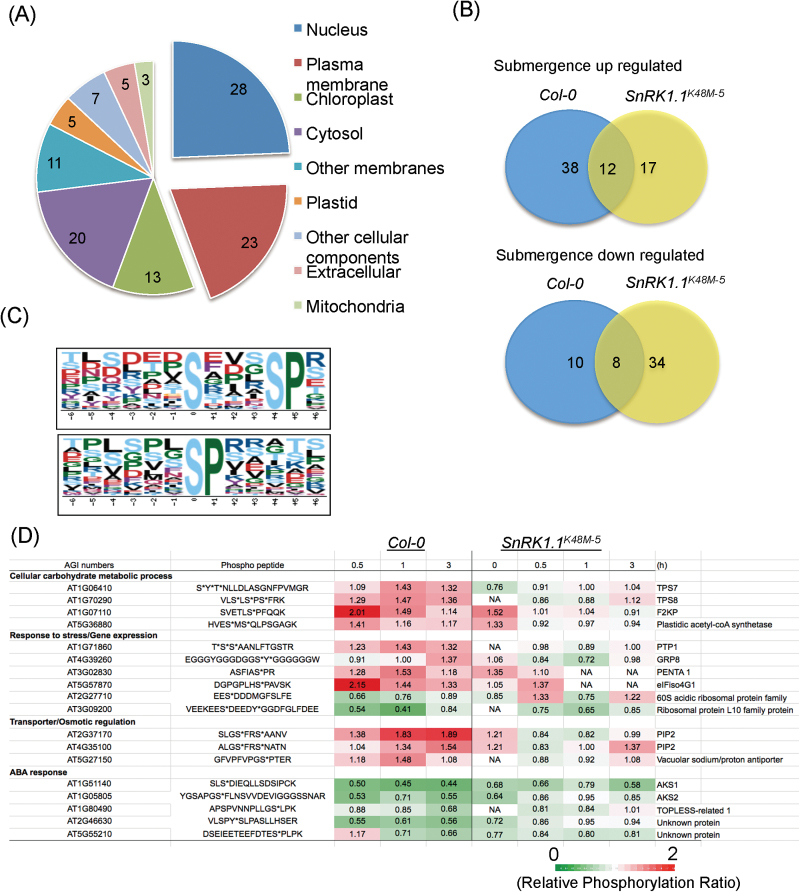
Phosphoproteomic profiles of the *SnRK1.1* ^*K48M*^ mutant and Col-0 under submergence. The proteins with phosphorylation levels that were significantly different at any time point under submergence (at room temperature in the dark) were selected to for analysis. (A) Gene ontology (GO) catalogues of the submergence-changed phosphorylated proteins in Col-0. (B) Venn diagram illustrating the overlap of up-regulated (upper diagram) and down-regulated (lower diagram) phosphorylated proteins identified in Col-0 and *SnRK1* ^*K48M*^. (C) Sequence logos of the phosphorylation sites seen in submergence up-regulated phosphopeptides. (D) The four gene ontology groups in the SnRK1.1 phosphorylation list, where the statistical differences (*P* value) of protein phosphorylation in treatment and control of Col-0 was smaller than 0.05, but that in *SnRK1* ^*K48M-5*^ was not;. ‘*’ represents the phosphorylation site. The numbers indicate the average value of the phosphorylation fold change of all the biological repeats. The time point data not detected in the three batches or only detected once in three batches is marked as NA (not available).

To determine the functions of SnRK1.1 under submergence, we applied iTRAQ to determine the changes in phosphorylation and the abundance of cellular proteins in Col-0 and *SnRK1.1* ^*K48M-5*^. The workflow of this analysis is illustrated in Supplementary Fig. S3. In total proteomics, 5 574 proteins were identified. In phosphoproteomics, the phosphorylation peptides with a pRS score (the possibility of phosphorylation in the peptide) ≥50 that were identified in two out of three repeats were selected. A total of 615 unique phosphopeptides that belong to 485 proteins was identified by phosphoproteomics (Supplementary Tables S1, S2). Three steps were used to generate a list of up- or down-regulated phosphopeptides from these 615 peptides. (i) We normalized all the data with the value of Col-0 in normal conditions. (ii) Because the standard deviations of three biological repeats were E0.3 (Supplementary Table S1), the proteins with an average fold-change in phosphorylation level >1.3 or <0.7 and that passed the bootstrap analysis (*P* <0.05) were selected as being significantly different under submergence. This generated 57 ‘***p***roteins with ***u***p-regulated ***p***hosphorylation’ (PUP) and 27 ‘***p***roteins with ***d***own-regulated ***p***hosphorylation’ (PDP) in Col-0 under submergence (Supplementary Tables S3, S4). These proteins were mainly located in the nucleus and plasma membrane (Fig. 3A) and were involved in carbohydrate metabolism (FDR 2.0E-05), gene expression (FDR 6.20E-05), nitrogen compound metabolism (FDR 5.5E-05), protein metabolism (FDR 1.60E-03), and transport (FDR3.4E-04) (Supplementary Tables S1, S3). We also identified 47 PUP and 60 PDP in SnRK1.1^K48M-5^ under submergence (Supplementary Table S1). (iii) Since we analysed the phosphorylated proteins profiles of Col-0 and *SnRK1.1* ^*K48M-5*^ by LC MS/MS separately, it meant that the phosphorylation of some proteins was only detected in one of the lines. Therefore, these proteins were excluded from the lists of PDP and PUP. In addition, some phosphorylated proteins in *SnRK1.1* ^*K48M-5*^ had pRS scores lower than 50, but not in Col-0, suggesting that their phosphorylation was disrupted by *SnRK1.1* ^*K48M-5*^. We added these phosphorylated proteins to the PUP lists. Taken together, there were 50 PUP and 18 PDP in Col-0 and 29 PUP and 42 PDP in *SnRK1.1* ^*K48M-5*^ under submergence (Fig. 3B).

Venn diagram analysis was then used to distinguish between SnRK1.1-dependent and -independent phosphorylation. The analysis of PUP in Col-0 and *SnRK1.1* ^*K48M-5*^ under submergence (Fig. 3B, upper panel; Supplementary Table S1) showed that 38 only appeared in Col-0, 12 appeared in both Col-0 and *SnRK1.1* ^*K48M-5*^, and 17 appeared only in SnRK1.1^K48M-5^. These results suggested that 55 PUP were under SnRK1 regulation. Noticeably, the protein levels of 38 ‘Col-0 only PUP’ were not changed significantly in Col-0 and *SnRK1.1* ^*K48M-5*^ under submergence (Fig. 3B; Supplementary Tables S5, S6), suggesting that SnRK1.1 only regulated their phosphorylation. On the other hand, in the analysis of PDP (Fig. 3B, lower panel; Supplementary Table S1), there were 10 PDP in Col-0 only, eight in both Col-0 and *SnRK1.1* ^*K48M-5*^, and 34 in *SnRK1.1* ^*K48M-5*^ only. These results suggested that the phosphorylation of 44 PDP was under SnRK1 regulation. The protein levels of 10 PDP in Col-0 only were not changed significantly in Col-0 and *SnRK1.1* ^*K48M-5*^ under submergence (Fig. 3B; Supplementary Tables S5, S6), revealing that SnRK1.1 was only involved in their phosphorylation reduction. Therefore, under submergence, 99 phosphorylated proteins, including 55 PUP and 44 PDP, were under SnRK1.1 regulation (Fig. 3B). In addition, two significantly enriched phosphorylation motifs were extracted from the lists of up-regulated phosphopeptides in Col-0 under submergence by the motif finding algorithm, Motif-X (Fig. 3C; Supplementary Table S7; Schwartz and Gygi, 2005). The second motif was also identified in the SnRK1.1-dependent phosphorylated proteins list, including TPS8 and F2KP, two well-known SnRK1 substrates.

### Identification of putative SnRK1.1 substrates

**Fig. 4.**
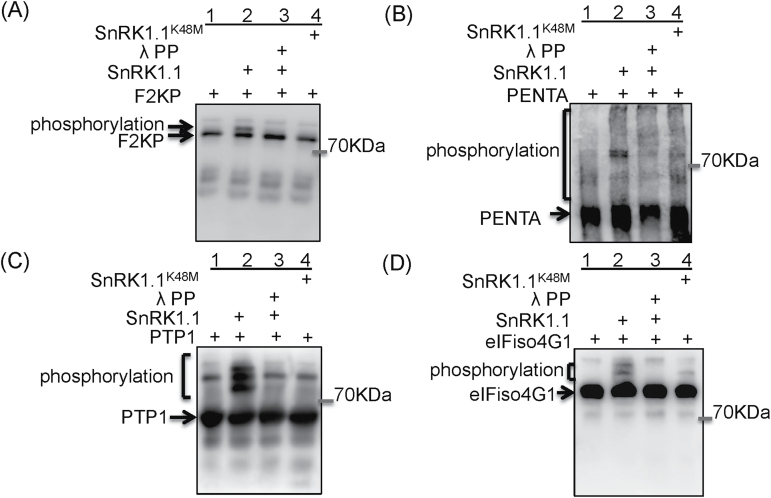
*In vitro* phosphorylation assays of SnRK1. The recombinant proteins fused with the S tag, (A) F2KP, (B) Penta, (C) PTP1, and (D) eIFiso4G1, were mixed with immunoprecipitated active SnRK1.1 (IP-SnRK1.1-S) or inactive SnRK1.1^K48M^ and the phosphorylation of these recombined proteins was examined with Phos-Tag page and S-tag immunoblotting. Phosphorylated protein mobility is retarded in Phos-Tag PAGE and this retardation can be reduced by the addition of λ phosphatase. Lane 1 of each blot was loaded with recombinant protein only, representing the mobility of non-phosphorylated substrate. In the second and fourth lanes of each blot, the four candidates showed mobile retardation in IP-active SnRK1.1 treatment but not in inactive SnRK1.1^K48M^ treatment. In the third lane of each blot, the retardation of the band shift was reduced with λ phosphatase treatment. At least three independent experiments were performed with similar results.

The majority of PUP under submergence was SnRK1.1-dependent, and their protein levels were not changed significantly in Col-0 and *SnRK1.1* ^*K48M-5*^ under submergence (Fig. 3D; Supplementary Tables S5, S6). To test whether they are direct targets of SnRK1.1, we selected four candidates in different functional categories, eIFiso4G1 (a translation initiation factor), PENTA (an RNA stability regulator), PTP1 (PROTEIN TYROSINE PHOSPHATASE 1, a primary target of ROS) (Supplementary Fig. S4A), and F2KP, which is a well-known SnRK1.1 substrate, for the *in vitro* kinase assay. These proteins were fused with His and S tags and were obtained by *E. coli* expression systems and affinity-purification. In addition, the active SnRK1.1 and inactive SnRK1.1^K48M^ were immunoprecipitated from Col-0 and *cMYC-SnRK1* ^*K48M-1*^ that had been submerged for 1h. The specificity of the IP products was examined by SnRK1.1 immunoblotting following Phos Tag SDS PAGE (Supplementary Figs S4B, S5). After mixing the four recombinant proteins with SnRK1.1 (IP-SnRK1.1-S) respectively, through S tag immunoblotting following Phos Tag SDS PAGE, the four recombinant proteins (Lane 2, Fig. 4A–D) showed mobility retardation when compared with the lanes in which only the recombinant proteins were loaded (Lane 1, Fig. 4A–D). To validate that the mobility retardation of these proteins was caused by SnRK1.1 phosphorylation, the mixture of IP-SnRK1.1-S and recombinant proteins were treated with λ phosphatase (Lane 3, Fig. 4A–D), or the recombinant proteins were mixed with inactive SnRK1.1^K48M^ (Lane 4, Fig. 4A–D). The mobility retardation of eIFiso4G1, PENTA, and PTP1 was shown only in the mixture of SnRK1, demonstrating that these three proteins were the substrates of SnRK1.1 *in vitro*.

### SnRK1 regulates anaerobic metabolism under submergence

**Fig. 5.**
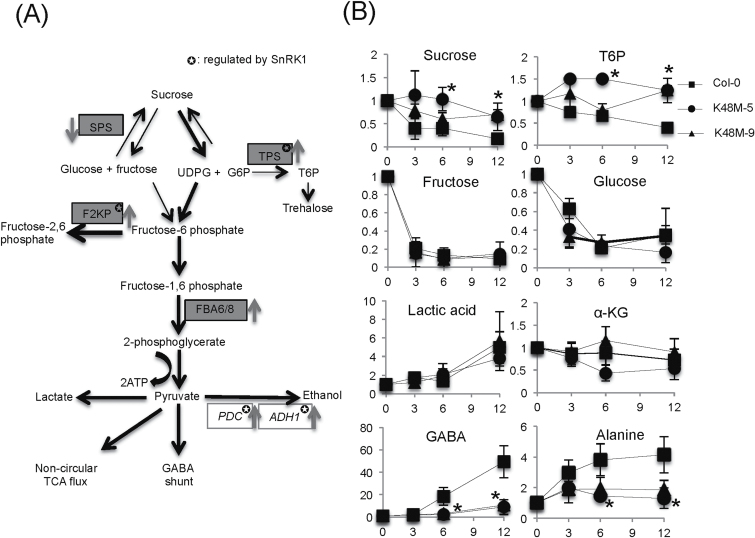
Effects of SnRK1.1 on the phosphorylation of glycolytic enzymes during submergence. (A) Schematic model of carbohydrate metabolism under submergence in Arabidopsis. Grey boxes indicate the gene under phosphorylation and white boxes represent the gene under transcriptional regulation. The arrows near the boxes show up- or down-regulation in Col-0 under submergence. Bold arrows indicate the promoting reaction under submergence. (B) The profile of soluble sugars, T6P, alanine, GABA, lactic acid, and α-KG in Col-0 and *SnRK1.1* ^*K48M*^ under submergence. The data represent means ±SD from three independent biological replicates. Statistical differences between Col-0 and mutant samples were determined by Student’s *t* test. *, *P* <0.05.

Two known SnRK1 substrates, F2KP and TPS7, had increased phosphorylation in Col-0, but were unchanged in *SnRK1.1* ^*K48M-5*^ under submergence (Supplementary Table S1). F2KP is involved in glycogenesis (Fig. 5A), and its phosphorylation by SnRK1 resulted in a decrease in its activity, leading to reduced energy consumption (Halford and Hey, 2009). TPS7, is a Class II TPS (Ramon *et al.*, 2009) and has been shown to have T6P binding affinity (Harthill *et al.*, 2006), but not clear TPS activity (Ramon *et al.*, 2009). T6P is an essential factor of metabolic signalling and an inhibitor of SnRK1.1 (Zhang *et al.*, 2009). T6P accumulation and the expression of Class II TPS are regulated by increased cellular sucrose (Ramon *et al.*, 2008). Thus, the Class II TPS protein is proposed to be involved in T6P sensing (Harthill *et al.*, 2006; Paul *et al.*, 2008). Interestingly, the reported phosphorylation site, the 5th serine of TPS7 recognized by SnRK1 and 14-3-3 *in vitro* (Harthill *et al.*, 2006) was also identified in our data (Supplementary Table S5). This phosphorylation site was conserved in TPS5, 6, and 7 and was phosphorylated in response to glycolysis inhibitor (2-deoxyglucose) that stimulates the activity of SnRK1 (Harthill *et al.*, 2006). In our data (Supplementary Table S1), the other Class II TPS, TPS8, also had increased phosphorylation and was also phosphorylated at the consensus sequence of SnRK1 (phiXXXX**S/T**XXXphi), which is a 10-residue motif with phi being a hydrophobic residue (M, L, V, I, or F), and X representing random amino acids (Halford and Hardie, 1998). These data indicated that, under submergence, SnRK1 might modulate metabolic signalling by phosphorylating these important metabolism factors.

Next, we quantified soluble sucrose and T6P in seedlings of Col-0, *SnRK1.1* ^*K48M-5*^, and *SnRK1.1* ^*K48M-9*^. Under submergence, plants initiate fermentation to regenerate NAD^+^. In addition to the fermentation products ethanol and lactic acid, plants also convert sucrose into alanine and alpha-ketoglutaric acid (α-KG). Alanine is accumulated, but α-KG is also used in the GABA shunt and non-circle TCA (Miyashita and Good, 2008).Therefore, we also quantified lactic acid, α-KG, alanine, and GABA in Col-0 and the two *SnRK1.1* ^*K48M*^ lines to examine the effect on glycolysis. Our data showed that sucrose and T6P were reduced in Col-0 in the early stage of submergence; however, there was a smaller decrease in sucrose and T6P in *SnRK1.1* ^*K48M-9*^ and no decrease in the stronger dominant negative mutant *SnRK1.1* ^K48M-5^ at the early stage (0–6h) of submergence (Fig. 5B). Glucose and fructose showed the same reduction patterns in Col-0 and *SnRK1.1* ^*K48M*^ under submergence (Fig. 5B), and the accumulation of lactic acid in Col-0 and *SnRK1.1* ^*K48M*^ was not significantly different, indicating that glucose and fructose were still being converted into lactic acid in *SnRK1.1* ^*K48M*^. Notably, the content of α-KG was not significantly changed in Col-0 and *SnRK1.1* ^*K48M*^ under submergence, but alanine and GABA were accumulated in Col-0, but were less accumulated in *SnRK1.1* ^*K48M-5*^ and *SnRK1.1* ^*K48M-9*^ (Fig. 5B). Accordingly, SnRK1.1 modulated sucrose and T6P degradation and altered GABA and alanine accumulation under submergence.

### SnRK1 communicates with MPK signalling under submergence

**Fig. 6.**
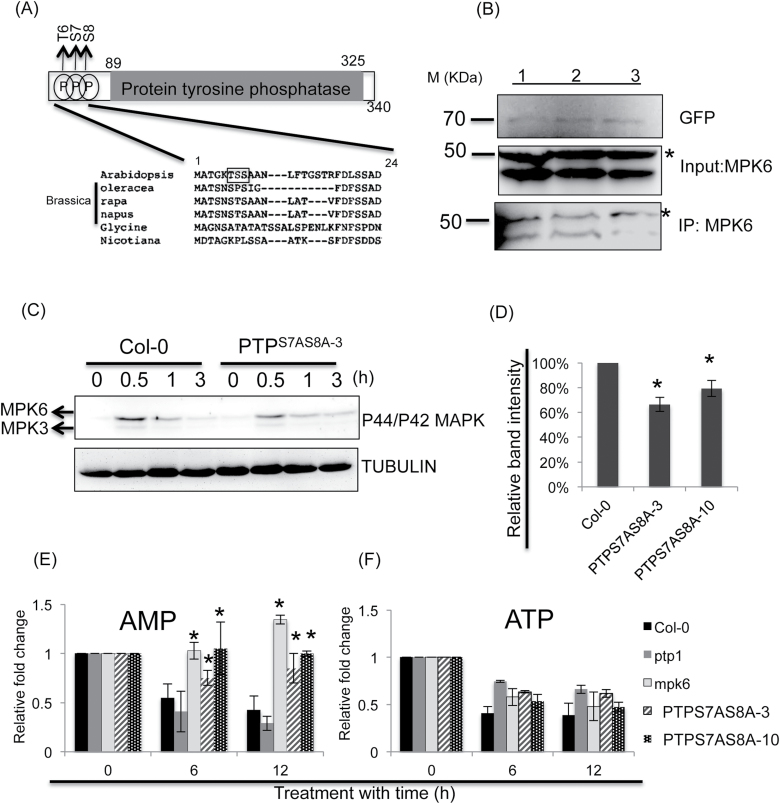
SnRK1.1 regulated MPK6 signalling by phosphorylating PTP1 under submergence. (A) Comparison of three phosphorylation sites (Thr6, Ser7, and Ser8) of Arabidopsis PTP1 with orthologues in other Brassica species, *Glycine max* and *Nicotiana tomentosiformis* by ClustalW. The black frame in the amino acid alignment indicates the phosphorylation sites of Arabidopsis PTP1. (B) The interaction of PTP1 and MPK6 was disrupted by PTP1^S7DS8D^. Upper panel: after immune precipitation, the abundance of (1) PTP1–GFP, (2) PTP^S7AS8A^–GFP, and (3) PTP1–^S7DS8D^–GFP conjugated in the beads of each transient samples. Middle panel: the abundance of MPK6 in the three PTP1 transient expression samples. Lower panel: the pull-down MPK6 were detected in PTP1–GFP and PTP^S7AS8A^–GFP transient expression samples, but not in PTP1–^S7DS8D^–GFP. *: Non-specific band. At least three biological repeats were performed and showed similar patterns. (C) Phosphorylation of MPK3/6 in Col-0, and *PTP1* ^*S7AS8A-3*^ under submergence. (D) The quantification of band intensity of (C) Western blot by GelEval software (FrogDance). The band intensity of 0.5h submergence in Col-0 and *PTP1* ^*S7AS8A*^ lines was quantified, and TUBULIN was used as an internal control. The AMP (E) and ATP (F) profiles in Col-0, *ptp1*, *mpk6*, and two *PTP1* ^*S7AS8A*^ lines under submergence. Statistical differences between Col-0 and mutant samples were determined by Student’s *t* test. *, *P* <0.05.

The other prominent group of putative SnRK1.1 substrates comprised proteins related to the stress response (Supplementary Table S5), including PROTEIN TYROSINE PHOSPHATASE 1 (PTP1). PTP1 is a primary target of H_2_O_2_ (Gupta and Luan, 2003) and a negative regulator of MPK3/6 activities (Bartels *et al.*, 2009). Recent studies showed that MPK3 and 6 are the central signalling components involved in different abiotic stresses (Taj *et al.*, 2010; Pucciariello *et al.*, 2012a). In the early stages of oxygen deprivation, MPK3 and 6 are activated by the interruption of the oxidative phosphorylation pathway in the mitochondria (Chang *et al.*, 2012). The level of phosphorylated PTP1 in *SnRK1.1* ^*K48M-5*^ is significantly lower than in Col-0 (Supplementary Table S5), and PTP1 was directly phosphorylated by SnRK1.1 *in vitro* (Fig. 4C; Supplementary Fig. S4C). Through MS-MS analysis, the phosphorylation sites of PTP1 by the *in vitro* kinase assay were identified at the 6th threonine (PTP^T6^) and 7th and 8th serine (PTP^S7^ and PTP^S8^) (Fig. 6A; Supplementary Table S1), which were also identified in our phosphoproteomics study (Supplementary Table S1). We compared the Arabidopsis PTP1 sequence with orthologues from other plants (Fig. 6A). Only the 7th amino acid of PTP1 in Brassicaceae and soybean (*Glycine max*) were serine or threonine, which can be phosphorylated. Interestingly, the phosphorylation possibility of PTP^S8^ is the highest in the *in vitro* kinase assay and two of three proteomic biological repeats. We therefore focused on the examination of phosphorylation of PTP1^S7^ and PTP^S8^.

Since MPK6 could interact with PTP1 (Gupta and Luan, 2003), we examined whether the phosphorylation of PTP1 interrupted its interaction with MPK6. We transiently expressed three forms of GFP-tagged constructs into the *ptp1* seedlings: wild-type PTP1–GFP; PTP1^S7AS8A^–GFP, in which two serines were replaced by alanines and could not be phosphorylated; and PTP1^S7DS8D^–GFP, which mimics phosphorylated PTP1,. Cellular extracts, with equal amounts of endogenous MPK6, were prepared from these seedlings and used for immunoprecipation of PTP1–GFP, PTP1^S7AS8A^–GFP, and PTP1^S7DS8D^–GFP. With the same amounts of endogenous MPK6 input (Fig. 6B, middle panel) and immnuoprecipitated PTP1–GFP, PTP1^S7AS8A^–GFP, and PTP1^S7DS8D^–GFP (Fig. 6B, upper panel), MPK6 could be pulled down by PTP1–GFP and PTP1^S7AS8A^–GFP (Fig. 6B, lanes 2 and 3, lower panel), but not by PTP1^S7DS8D^ –GFP (Fig. 6B, lane 3, lower panel). This indicated that the interaction of PTP1 and MPK6 was disrupted by phosphorylation of the 7th serine and 8th serine of PTP and the interaction of PTP1–GFP with MPK6 was not caused by the GFP fusion tag. These results suggested that, through phosphorylating PTP1, SnRK1 disrupted the interaction of PTP1 and MPK6 to enhance the MPK6 signalling.

To examine this hypothesis, we transformed *PTP1* ^*S7AS8A*^ into *ptp1* to generate transgenic lines and two transgenic lines were selected that showed expression levels of *PTP1* close to those of Col-0 (Supplementary Fig. S6A). We compared the MPK3/6 phosphorylation in Col-0, *ptp1*, and the two *PTP1* ^*S7AS8A*^ lines. MPK6 was phosphorylated in Col-0 after 30min of submergence, but the phosphorylation of MPK3 was not significant (Fig. 6C). The increases in phosphorylation levels of MPK6 in *ptp1* and Col-0 were not significantly different (Supplementary Fig. S6B). However, the increases in MPK6 phosphorylation in the two *PTP1* ^*S7AS8A*^ transgenic lines were lower than in Col-0. (Fig. 6C, D), indicating that non-phosphorylated *PTP1* ^*S7AS8A*^ could prevent MPK6 from being phosphorylated during submergence. To validate the impact of MPK6 phosphorylation in *PTP1* ^*S7AS8A*^ under submergence, we also compared the cellular levels of AMP and ATP in the two *PTP1* ^*S7AS8A*^ lines*, mpk6, ptp1*, and Col-0 (Fig. 6E). The ATP was reduced in all of these five lines, demonstrating that energy reduction caused by oxygen deprivation was not impacted by MPK6 and PTP1. Interestingly, AMP was reduced in Col-0 and *ptp1* after 6h submergence, but AMP was accumulated more in *mpk6* and was not significantly reduced in the two *PTP1* ^*S7AS8A*^ lines. These results suggested that MPK6 was involved in energy conservation under submergence and PTP1 ^*S7AS8A*^ interfered with MPK6 signalling by preventing MPK6 phosphorylation.

### SnRK1.1 interacts with MPK6 and PTP1

**Fig. 7.**
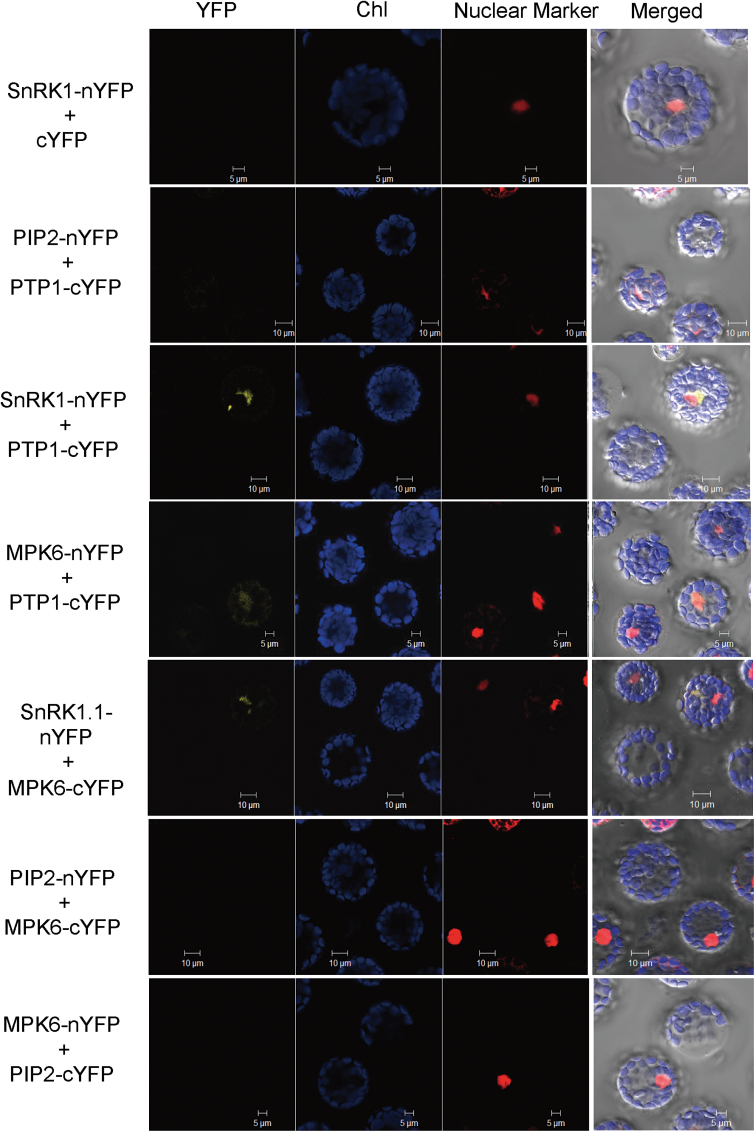
Protein–protein interactions between PTP1, SnRK1.1, and MPK6. Examination of the interactions between PTP1 and SnRK1.1, PTP1 and MPK6, and MPK6 and SnRK1.1 in protoplasts by BiFC. SnRK1.1-nYFP+cYFP, MPK6-nYFP+PIP2-cYFP, MPK6-cYFP+PIP2-nYFP, and PTP1-cYFP+PIP2-nYFP were the negative controls. The images have different interference contrasts: (lane 1) YFP fluorescence by BiFC, (lane 2) chloroplast auto fluorescence (Chl), (lane 3) mCherry fluorescence as a nuclear marker and transient effect control, and (lane 4) merged image.

We performed bimolecular fluorescence complementation (BiFC) assays to examine the interaction of SnRK1.1, PTP1, and MPK6. The C-terminal region of *Agobacterium tumefaciens* Vird2 containing the bipartite nuclear localization signal (NLS) fused with mCherry was used as a nuclear marker and a transient effect control (Fig. 7, lane 3) (Wu *et al.*, 2009). To examine the specificity in BiFC, the pairs of SnRK1.1-nYFP and vector 3130 (pSAT-35S::cYFP-DEST), MPK6-nYFP and PIP2-cYFP, MPK6-cYFP and PIP2-nYFP, PTP1-cYFP and PIP2-nYFP were co-transfected into protoplasts, respectively, as a negative control (Fig. 7, panels 1, 2, 6, 7). On the other hand, three pairs, MPK6-nYFP and PTP1-cYFP, SnRK1.1-nYFP and PTP1-cYFP, and SnRK1.1-nYFP and MPK6-cYFP (Fig. 7, panels 3, 4, 5) were transfected into protoplasts, respectively. The results demonstrated that SnRK1.1 mainly interacts with PTP1 or MPK6 in the cytoplasm (Fig. 7, lane 4, panels 3, 5), but PTP1 interacted with MPK6 in the cytoplasm and nucleus (Fig. 7, lane 4, panel 4). We also examined the interaction of MPK6 with PTP1^S7AS8A^ or PTP1^S7DS8D^. However, we could not distinguish the interaction of these proteins, due to the high background signal of PTP1^S7AS8A^ and PTP1^S7DS8D^ with the negative control (Supplementary Fig. S7). Taken together, these results suggested that SnRK1.1 directly interacted with PTP1 to phosphorylate PTP1.

### SnRK1.1 regulates MPK6 signalling

**Fig. 8.**
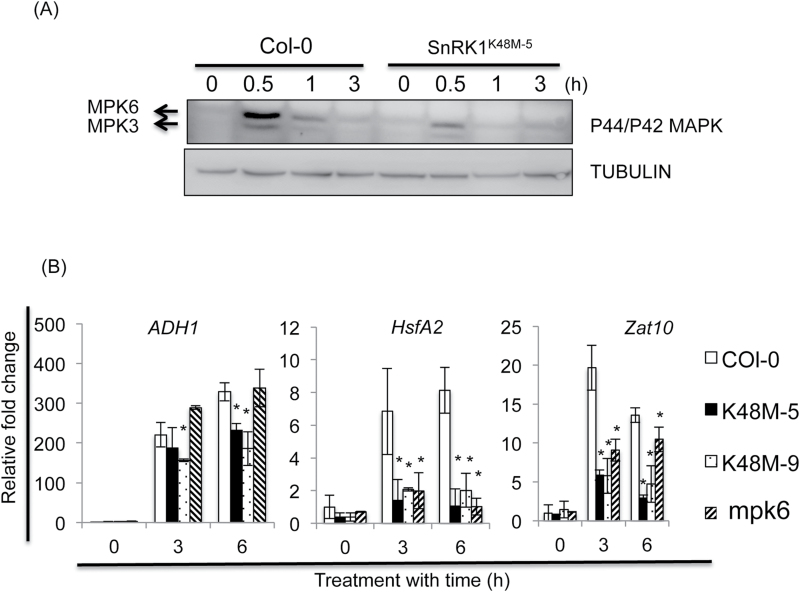
SnRK1.1 regulated the MPK6 signalling pathway under submergence. (A) Phosphorylation of MPK3 and MPK6 in Col-0 and *SnRK1.1* ^*K48M-5*^ under submergence. The data represent three independent biological replicates. TUBULIN was used as an internal control. (B) Expression of MPK signalling marker genes in Col-0, *SnRK1.1* ^*K48M*^, and *mpk6*. Transcript levels were detected by qRT-PCR by specific primers. *TUBULIN* mRNA was the internal control. The data represent means ±SD from four independent biological replicates. Statistical differences between Col-0 and the mutants were determined by Student’s *t* test. *, *P* <0.05.

To determine whether SnRK1 regulates MPK6 signalling, we compared the phosphorylation of MPK6 in Col-0 and *SnRK1.1* ^*K48M-5*^ (Fig. 8A). Notably, the level of phosphorylated MPK6 was lower in *SnRK1* ^*K48M*^ than in Col-0 under submergence (Fig. 8A). The MPK6 protein abundance was not affected in *SnRK1* ^*K48M*^ (Supplementary Tables S2, S6). To validate that MPK6 signalling is affected in *SnRK1* ^*K48M*^ mutants, we compared the expression of predicted downstream targets of MPK6, *HsfA2* and *ZAT10* (Pucciariello *et al.*, 2012a), in Col-0, *SnRK1.1* ^*K48M-5*^, and *mpk6* under submergence. Both *HsfA2* and *ZAT10* were induced in Col-0 after 3h and 6h of submergence (Fig. 8B). However, the expression of *HsfA2* and *ZAT10* were significantly lower in *SnRK1.1* ^*K48M*^ and *mpk6* than in Col-0 under submergence. In addition, *ADH1* had a lower induction level in the two *SnRK1.1* ^*K48M*^ mutants than in Col-0, but had a similar level in *mpk6* as in Col-0 under submergence (Fig. 8B). Taken together, the results shown in Figs 6–8 indicate that SnRK1.1 enhanced MPK6 signalling by disrupting the interaction of PTP1 and MPK6 under submergence.

## Discussion

### SnRK1 is a master regulator of anaerobic metabolism under submergence

SnRK1 has a central role in sugar signalling pathways (Jossier *et al.*, 2009). Two important sugar metabolic enzymes, TPS and F2KP, are phosphorylated by SnRK1 *in vitro* (Halford and Hey, 2009). We have reported here that these two enzymes are phosphorylated via SnRK1.1 in Arabidopsis under submergence. We have shown that the activity of SnRK1.1 was triggered under submergence and the inactive form of SnRK1.1 disrupted the endogenous SnRK1.1 to phosphorylate its targets. By measuring the cellular levels of various metabolites in Col-0 and *SnRK1.1* ^*K48M*^, we have shown that SnRK1 enhances sucrose and T6P degradation, and alanine and GABA accumulation under submergence (Fig. 5B). The reduction of T6P, a negative regulator of SnRK1 activity (Delatte *et al.*, 2011), suggested that SnRK1.1 might repress T6P synthesis to maintain its activity by phosphorylating TPS7 and 8. However, these TPSs did not have a clear TPS function under normal conditions (Ramon *et al.*, 2009). It will be interesting to know whether they play a role in T6P metabolism under submergence. In addition, one of the SnRK1 targets, F2KP, is a bifunctional enzyme that can generate fructose-6-phosphate or fructose-2,6-bisphosphate. Fructose-2,6-bisphosphate is accumulated under hypoxia and has been proposed to be a signalling molecule for regulating glycolysis (Mertens *et al.*, 1990; Nielsen *et al.*, 2004). Collectively, our results suggest that SnRK1 might phosphorylate F2KP to switch its activity to produce fructose-2,6-bisphophate and then enhance the glycolysis pathway to adapt to energy starvation.

### SnRK1 modulates MPK signalling under submergence

**Fig. 9.**
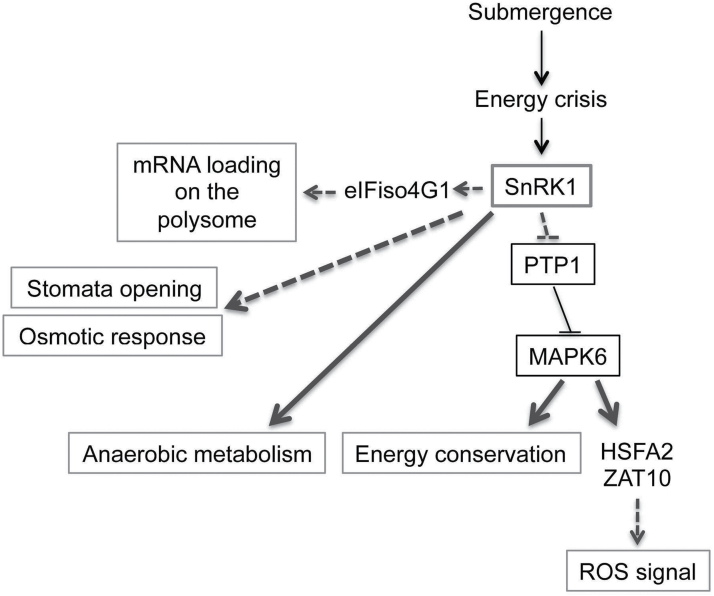
Summary of SnRK1.1-mediated pathways under submergence. Solid lines indicate validated events and bold lines represent the new signalling pathways mediated by SnRK1 under submergence. Dotted lines indicate the predicted events.

MPK6 signalling is a general stress signal whose phosphorylation can be triggered by the ROS and MKK cascades (Taj *et al.*, 2010). Interestingly, ROS is an important signal that can enhance ethylene signalling and *ADH1* expression during oxygen deprivation (Pucciariello et al., 2012*a*, *b*; Yang, 2014), and MPK6 is the predominant MPK regulated by ROS released from the disruption of the electron transportation chain in the mitochondria during oxygen deprivation and re-oxygenation. We also showed that the induction levels of two ROS responsive marker genes, *HsfA2* and *ZAT10* (Pucciariello et al., 2012*a*, *b*), were reduced in *mpk6* under submergence. Transgenic lines that overexpress *MPK6* are more tolerant than Col-0 under submergence (Chang *et al.*, 2012). These results suggested that the ROS response mediated by MPK6 signalling might contribute to improve submergence tolerance. Interestingly, under submergence, MPK6 phosphorylation was reduced in the *SnRK1.1* mutant and *PTP1* ^*S7AS8A*^ by the phospho p44/p42 MAPK antibody. This antibody recognizes the (TXY) motif of MPK6 phosphorylation, revealing that the phosphorylation of MPK6^T221^ and MPK6^Y223^ was disrupted by PTP1 and SnRK1.1 under submergence. The TXY (X-D/E/P) motif is a dual phosphorylation site and phosphorylation of both residues is essential for the activation of the MPKs (Taj *et al.*, 2010). PTP1 is a negative regulator dephosphorylating the tyrosine phosphorylation of MPK6 (Gupta and Luan, 2003), suggesting that PTP1 regulated MPK6^Y223^ phosphorylation under submergence. To address how SnRK1.1 disrupted MPK6 signalling by phosphorylating PTP1, we demonstrated that MPK6 could interact with PTP1, but not with PTP1^S7DS8D^, which mimics the phosphorylated PTP1 (Fig. 6B). Although we could not directly examine the disruption of the interaction of PTP1 with MPK6 by the phosphorylation of PTP1 under submergence, the reduction of MPK6 phosphorylation in two *PTP1* ^*S7AS8A*^ lines (Fig. 6C, D) and the maintenance of AMP in *mpk6* and two *PTP1* ^*S7AS8A*^ lines (Fig. 6E) supported that PTP1 phosphorylation by SnRK1.1 was required for MPK6 signalling in energy conservation under submergence. In addition, energy conservation (Fig. 2E), MPK6 phosphorylation, and signalling were also interrupted in the *SnRK1.1* mutants (Fig. 8). Accordingly, we developed a possible signalling cascade that SnRK1 enhances the MPK6 signal through disrupting the interaction of PTP1 and MPK6 under submergence (Fig. 9).

### SnRK1 regulates protein synthesis under submergence

Protein synthesis is an extremely energy-consuming process. In Arabidopsis cells under oxygen deprivation, in order to conserve energy, specific mRNAs are selected for translation into proteins (Branco-Price *et al.*, 2008) or for storage in stress granules (Sorenson and Bailey-Serres, 2014). However, little is known about how the mRNAs are selected for translation in Arabidopsis. In animals, protein synthesis is regulated by the phosphorylation of specific translation initiation factors and ribosomal subunits. AMPK, the orthologue of SnRK1 in animals could phosphorylate mTOR which, in turn, phosphorylated the eIF4 complex to repress translation under hypoxia (Shimobayashi and Hall, 2014). In Arabidopsis, the TOR complex and S6K1 promote translation initiation (Moreau *et al.*, 2010; Schepetilnikov *et al.*, 2013). Notably, through identification of the targets of SnRK1.1, our data also revealed that SnRK1 modulates the phosphorylation of a specific eIF4 subunit under submergence (Fig. 4D; Supplementary Tables S1, S5). It is known that eIF4A is phosphorylated within 20min of anoxia in maize and that eIF4A is an ATP dependent RNA helicase and regulates the eIF4G–eIF3 complex turnover in yeast (Webster *et al.*, 1991; Munoz and Castellano, 2012). In Arabidopsis under submergence, the cellular ATP content decreased dramatically (Fig. 2E) suggesting that the eIF4Fcomplex is involved in translation regulation under submergence. However, plants have two translation initiation complexes, eIF4F and eIFiso4F, to modulate differential translations of plant mRNAs (Mayberry *et al.*, 2009; Patrick and Browning, 2012). It will be interesting to characterize the roles of these two eIF4F complexes and whether SnRK1.1 is involved in regulating translation initiation through phosphorylating these translation initiation factors under submergence.

On the other hand, two known RNA binding proteins, PENTA and GRP8, were found to be regulated by SnRK1. PENTA is known to bind to the 5′ UTR region of protochlorophyllide reductase (*PORA*) mRNA to regulate mRNA stability (Paik *et al.*, 2012), and GRP8 is involved in the RNA spliceosome to alter the RNA splicing (Schoning *et al.*, 2008). Among these candidates, eIFiso4G1 and PENTA were identified as the direct targets of SnRK1.1 (Fig. 4B, D). These findings suggest that SnRK1 may be involved in protein synthesis regulation under submergence.

### SnRK1 synergistically regulates ABA signalling under submergence

SnRK1.1 is known to be a central regulator of the ABA and sugar signalling pathways. ABA is involved in systemic responses under flooding (Hsu *et al.*, 2011), but is degraded under submergence in Arabidopsis (Benschop *et al.*, 2007; Hsu *et al.*, 2013). To examine whether SnRK1.1 is involved in ABA signalling under submergence, we compared the phosphoproteomic profiles of submergence with another phosphoproteomics analysis of ABA treatment (Fig. 3D; Kline *et al.*, 2010). The phosphorylation of NHX1 and two members of the plasma membrane intrinsic protein (PIP) family of aquaporins were increased in Col-0, but not in *SnRK1.1* ^*K48M*^ under submergence (Fig. 3D; Supplementary Table S5). PIP2 has been proposed to be an osmosensor (Maurel *et al.*, 2008) and a cytosolic pH sensor in the roots (Tournaire-Roux *et al.*, 2003). The phosphorylated PIP2^S283^ is transported to intracellular spherical bodies (Li *et al.*, 2011) and controlled by ABA and salt stress (Kline *et al.*, 2010). Here, our data also suggested that SnRK1.1 might trigger the phosphorylation of PIP2^S283^ to re-localize PIP2 in the intracellular spherical bodies in order to reduce water flow in cells under submergence.

In addition, AKS1 and AKS2, which are involved in ABA-dependent stomata closure (Takahashi *et al.*, 2013), were dephosphorylated in Col-0, but remained phosphorylated in *SnRK1* ^*K48M*^ under submergence (Supplementary Table S5), indicating that SnRK1 might be involved in regulating stomatal closure under submergence. Stomatal closure has been proposed to be a part of the hypoxia-triggered immunity response (Hsu *et al.*, 2013) and to be involved in O_2_ influx into the shoots under submergence. Under submergence, there is a balance between O_2_ influx and consumption in the shoots. Plants can maintain the O_2_ level in the shoots by taking up O_2_ from stomata during dark periods or photosynthesis; it is then diffused to the roots through gas space diffusion (Lee *et al.*, 2011). These findings, together with our results, raise the possibility that SnRK1 and ABA synergistically regulate stomatal opening to diffuse O_2_ into the shoots in the early stage of submergence.

In summary, through our phosphoproteomic analysis, we discovered many SnRK1.1 downstream targets that participate in various biological processes (Fig. 9). Among these, proteins involved in the hypoxic response and the sugar metabolism enzymes, TPS and F2KP, have been well characterized in the SnRK1-mediated energy starvation response. The majority of these proteins still need to be examined further to dissect the significance of their phosphorylation under submergence. We started to investigate the potential significance of phosphorylation of the ROS signal regulator, PTP1, which is a negative regulator of MPK6. Examination of downstream targets of PTP1 indicated that SnRK1 might be involved in PTP1-MPK6 signalling and the downstream transcriptional regulation. Considering that the energy starvation response and ROS are critical signals of oxygen deprivation, our results show that SnRK1-mediated phosphorylation is important in the early stage of submergence.

## Supplementary data

Supplementary data are available at *JXB* online.

Figure S1. The heterocomplex profile of SnRK1 under submergence.

Figure S2. The profile of SnRK1 transgene lines.

Figure S3. Flowchart of iTRAQ sample preparation and prediction of SnRK1.1 targets.

Figure S4. The recombinant proteins of SnRK1.1 substrates and the phosphorylation pattern of IP-SnRK1.1 under submergence.

Figure S5. Immunoprecipitation of SnRK1 in Col-0 and inactive SnRK1^K48M^ in *cMYC-SnRK1.1* ^*K48M*^.

Figure S6. The profile of two PTP1 transgene lines.

Figure S7. Examination of the interactions between PTP1^S7AS8A^ and PTP1^S7DS8D^ with negative controls.

Table S1. Quantification of phosphoproteins in Col-0 and *SnRK1.1* ^*K48M*^ under submergence.

Table S2. Quantification of proteins in Col-0 and *SnRK1.1* ^*K48M*^ under submergence.

Table S3. Phosphorylation peptides up-regulated in Col-0 under submergence.

Table S4. Phosphorylation peptides down-regulated in Col-0 under submergence.

Table S5. Differential phosphorylation of selected submergence- responsive genes in *SnRK1.1* ^*K48M*^ mutants.

Table S6. Protein abundance of differential phosphorylation responsive genes in *SnRK1.1* ^*K48M*^ mutants.

Table S7. Phosphorylation motifs identified in whole seedings of Arabidopsis under submergence.

Table S8. Primer list.

Supplementary Data
