# Supplementary material for: Quantitative phosphoproteomics of protein kinase SnRK1 regulated protein phosphorylation in Arabidopsis under submergence
Source: J Exp Bot. 2016 Mar 29;67(9):2745–60. doi: 10.1093/jxb/erw107 (PMC4861021; doi:10.1093/jxb/erw107)
Supplement: Supplementary Data [file supp_67_9_2745__index.html]

Quantitative phosphoproteomics of protein kinase SnRK1 regulated protein phosphorylation in Arabidopsis under submergence — Quantitative phosphoproteomics of protein kinase SnRK1 regulated protein phosphorylation in Arabidopsis under submergence — Supplementary Data 

# Quantitative phosphoproteomics of protein kinase SnRK1 regulated protein phosphorylation in Arabidopsis under submergence

## Supplementary Data

Data files

- supplementary\_table\_S1.xlsx - Supplementary Data
- supplementary\_table\_S2.xlsx - Supplementary Data
- supplementary\_figures\_S1\_S7\_tables\_S3\_S8.pdf - Supplementary Data
