# Supplementary material for: Quantitative phosphoproteomics of protein kinase SnRK1 regulated protein phosphorylation in Arabidopsis under submergence
Source: J Exp Bot. 2016 Mar 29;67(9):2745–60. doi: 10.1093/jxb/erw107 (PMC4861021; doi:10.1093/jxb/erw107)
Supplement: Supplementary Data [file supp_erw107_supplementary_figures_S1_S7_tables_S3_S8.pdf]

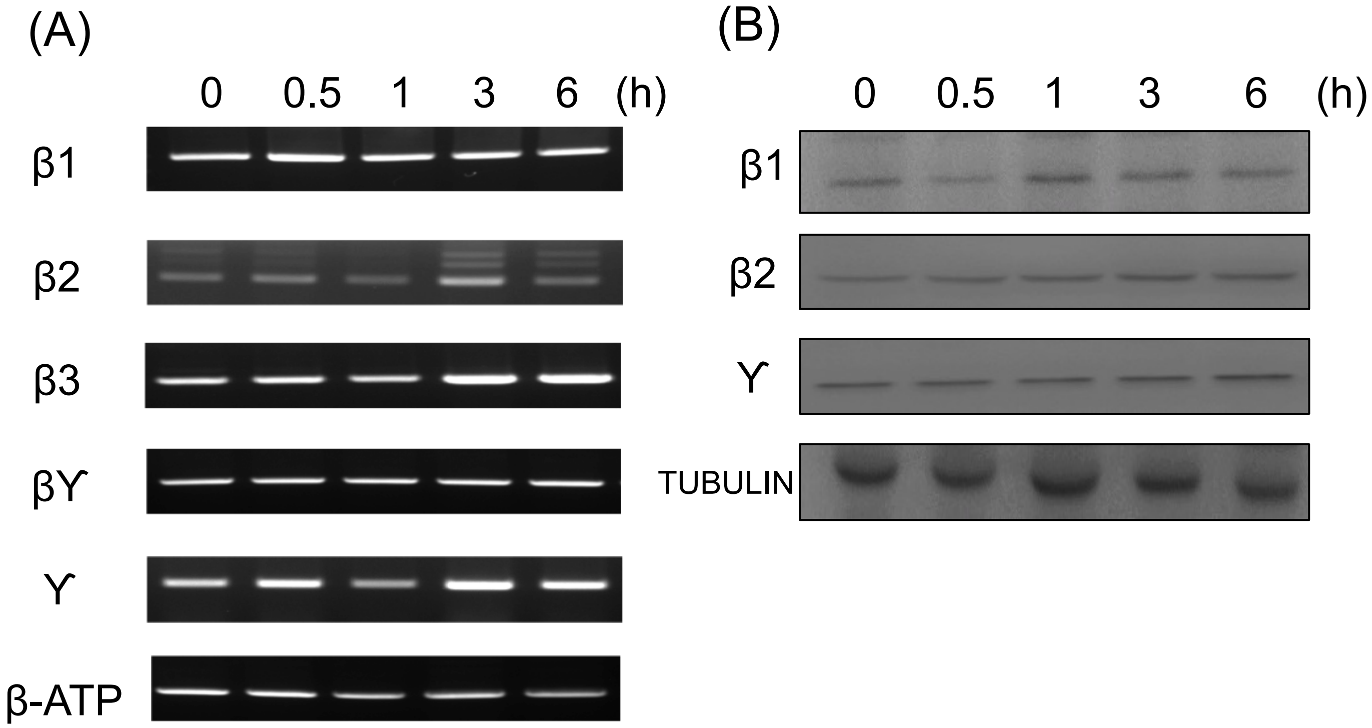

Fig. S1 The profile of heterocomplex of SnRK1 under submergence. The transcriptional level (A) and the protein abundance (B) of SnRK1 heterocomplex at different durations of submergence. The western blot experiments were analyzed by anti-akin β1, anti-akin β2, and anti- ϒ (Agrisera). β-*ATP* and TUBULIN were used as internal control in (A) and (B), respectively.

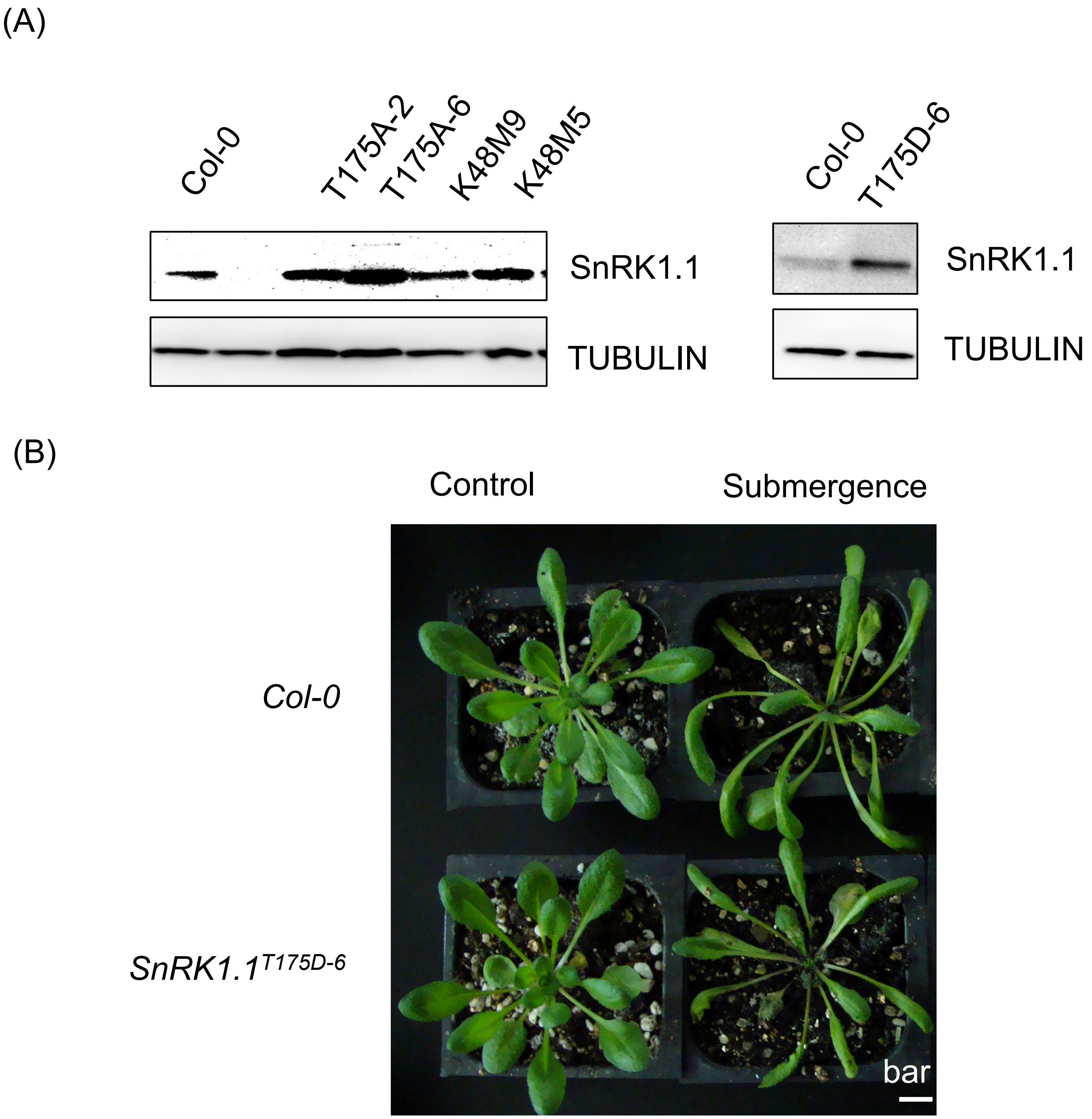

Fig. S2 The profile of SnRK1 transgene lines. (A) The profile of SnRK1 protein abundance of SnRK1^{T175A}, SnRK1^{T175D}, and SnRK1^{K48M}. TUBULIN was used as an internal control. (B) The phenotype of SnRK1.1$^{T175D-6}$ and *Col-0,* after submergence for 60 h in the adrk at 22°C and recovery for 4 days. Bar = 1 cm.

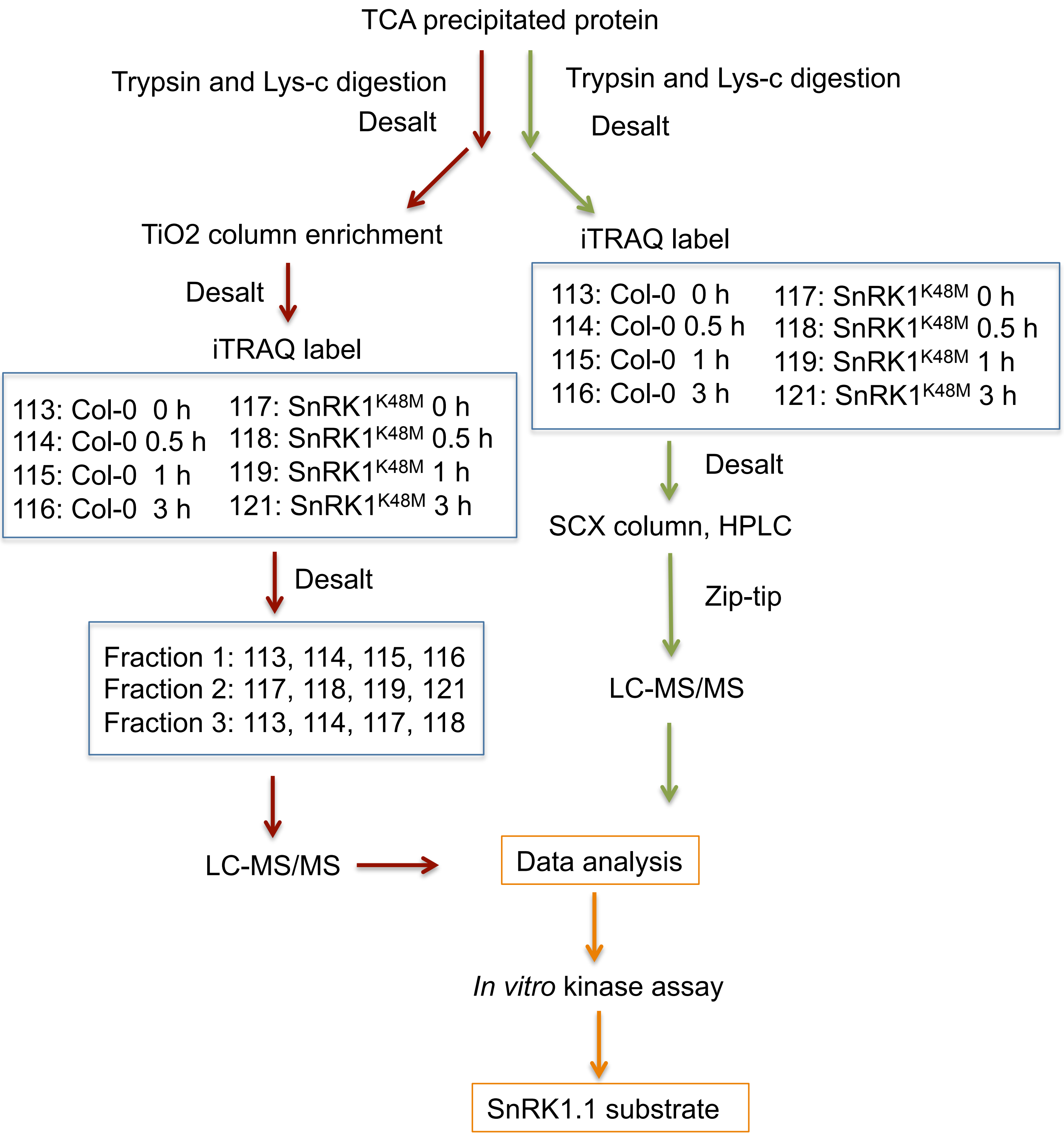

Fig. S3 Flowchart of iTRAQ sample preparation and prediction of SnRK1.1 targets. Total proteins of 9-day-old seedlings with different durations of submergence treatment were TCA precipitated and resuspended in the Tris buffer. They were further processed and fractionated for total proteomics or phosphoproteomics. The processes indicated by red and green arrows were for sample preparations in phosphoproteomics and total proteomics, respectively. After LC-MS/MS, the data were analyzed by the Proteome Discoverer software (ver. 1.3, Thermo Scientific) to generate the list of SnRK1.1-dependent phosphorylated protein under submergence. To identify the SnRK1.1 potential targets, the proteins identified in the SnRK1.1 dependent list were purified and examined their phosphorylation by the SnRK1.1 using the *in vitro* kinase assay.

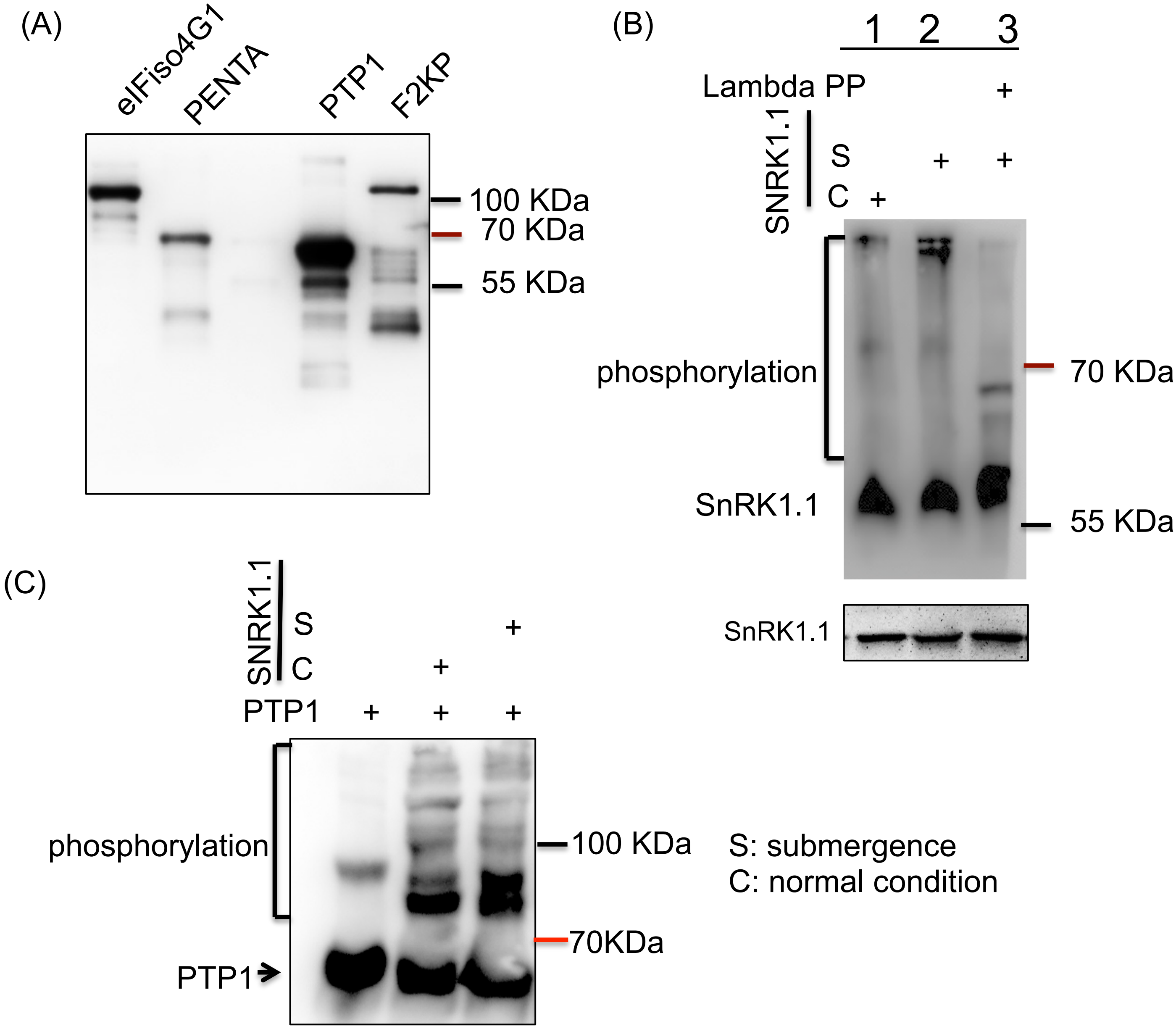

Fig. S4 The recombinant proteins of SnRK1.1 substrates and phosphorylation pattern of IP-SnRK1.1 under submergence. (A) The expressed and purified recombinant proteins, PTP1, F2KP, eIFiso4G1, and PENTA were examined by S-tag immunoblot. (B) Examination of the immunoprecipitated SnRK1.1 (IP-SnRK1.1) phosphorylation through Phos Tag SDS PAGE. To examine the phosphorylation profile of IP-SnRK1.1 under submergence, the IP-SnRK1.1 under different treatments was load in the SDS page (Panel 2) and Phos Tag SDS PAGE (Panel 1) and then detected by SnRK1.1 immunoblotting. In Panel1, the band at the bottom of blot was non-phosphorylated SnRK1.1. Comparing with IP-SnRK1.1 in the normal condition (Panel1, lane 1), different phosphorylated forms of SnRK1.1 were detected under submergence (Panel 1, lane 2) in the same amount of IP-SnRK1.1 loading (Panel 2). λ phosphatase treatment (lane 3) confirmed that the higher band shifting in lane 2 was caused by SnRK1.1 phosphorylation. (C) *In vitro* kinase assay of PTP1. The recombinant proteins fused S tag of PTP1 was mixed with immunoprecipitated SnRK1.1 under normal conditions (IP-SnRK1.1-C) or SnRK1.1 under submergence (IP-SnRK1.1-S), and the phosphorylation of PTP1 was examined through Phos-Tag page and S-tag immunoblot. The recombinant protein-only was loaded in the first lane, representing the mobility of non-phosphorylated substrate. In the second and third lanes, PTP1 showed mobile retardation in IP-SnRK1.1-S and IP-SnRK1.1-C. At least three independent experiments were performed with similar results.

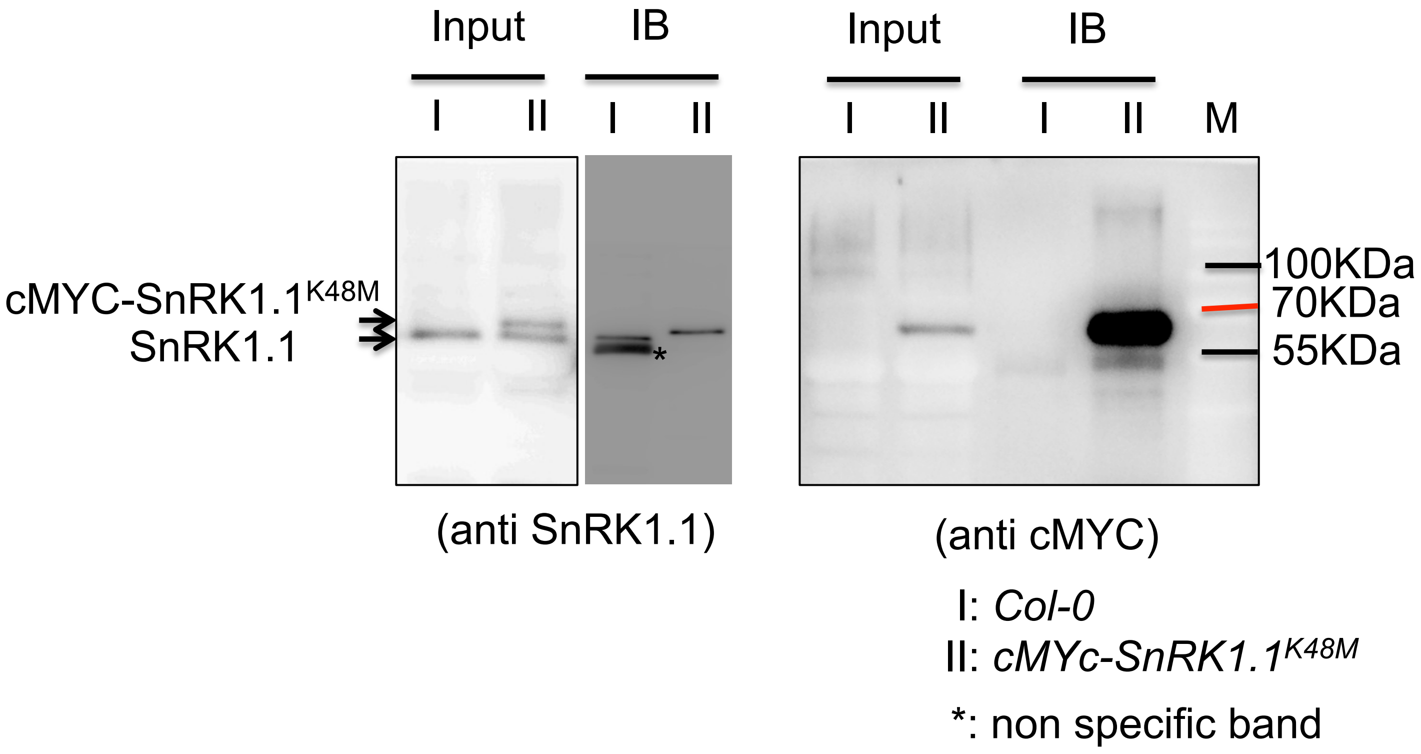

Fig. S5 Immunoprecipitation of SnRK1 in *Col-0* and inactive SnRK1^{K48M} in *cMYC-SnRK1.1^{K48M}.* The input is the total protein extracted from (I) *Col-0* and (II) *cMYC-SnRK1.1^{K48M}*. The IB represents the precipitation product of SnRK1.1 antibody in *Col-0* or cMYC antibody in *cMYC-SnRK1.1^{K48M}*.

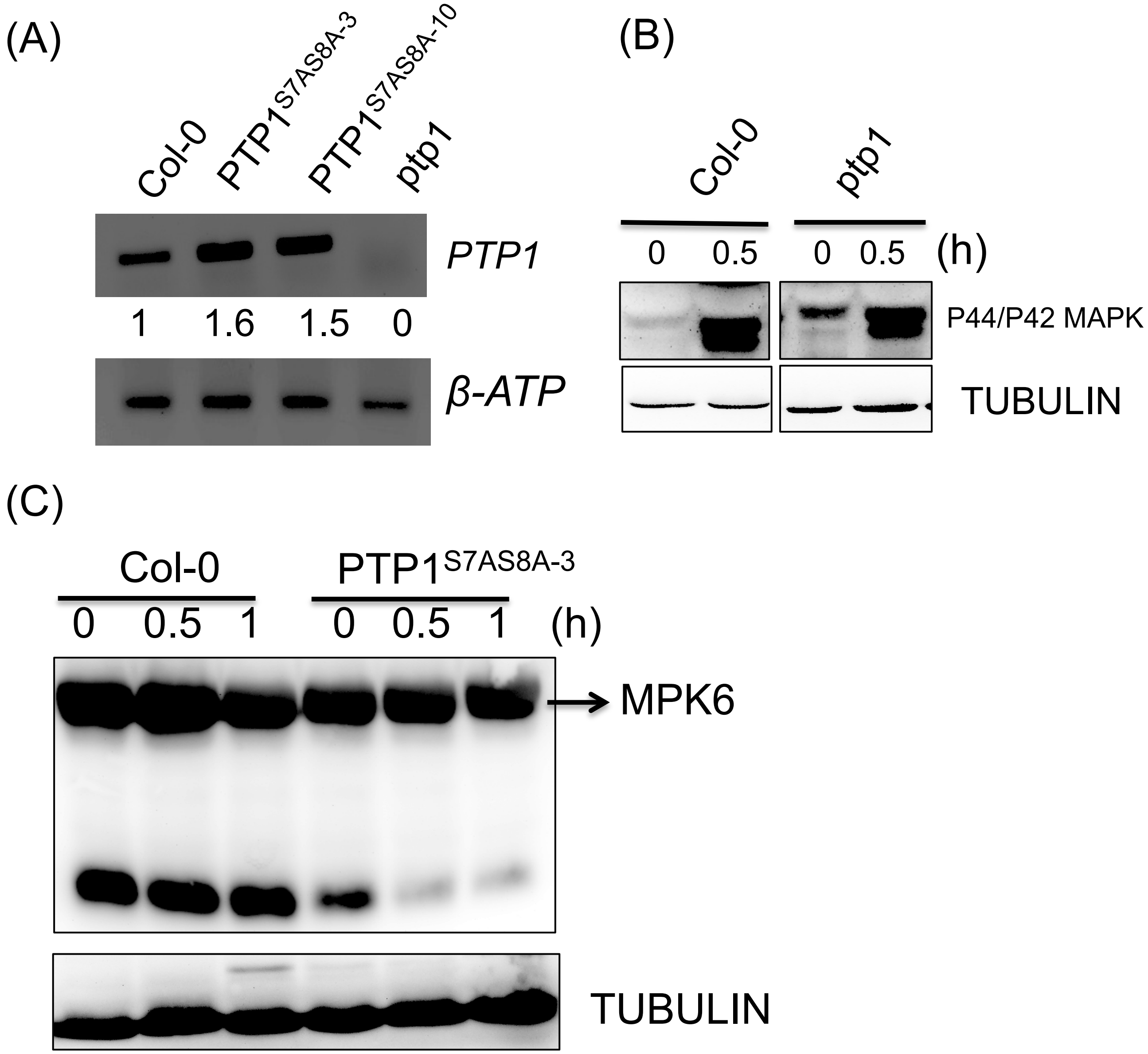

Fig. S6 The profile of two PTP1 transgene lines. (A) The quantification of *PTP1* expression in two *PTP1*^{S7AS8A} transgene lines, *Col-0*, and *ptp1*, by RT-PCR. The second panel represents the relative band intensity quantified by GelEval software (FrogDance), which was normalized by the band intensity of *β-ATP*. (B) The phosphorylation of MPK6 in *Col-0* and *ptp1* under submergence. (C) The MPK6 abundance in *Col-0* and *PTP1*$^{S7AS8A-3}$ under submergence. The data represent three independent biological replicates. Tubulin was used as an internal control.

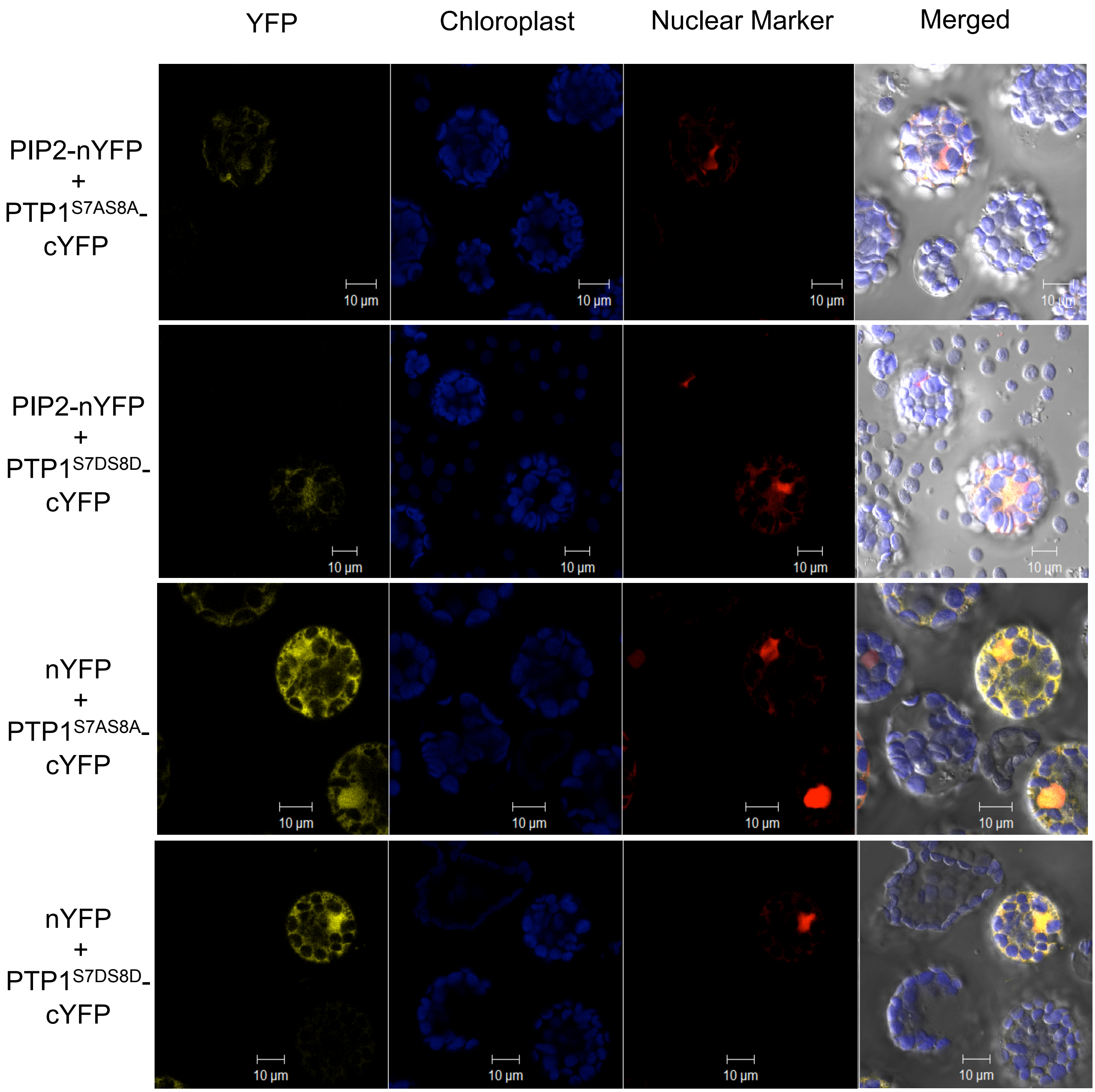

Fig. S7 Examination of the interactions between PTP1[S7AS8A] and PTP1[S7DS8D] with negative controls. Empty vector (35S::nYFP) or PIP2-nYFP was used as the negative control of BIFC.

Supplemental materials and methods

ATP and AMP assay

Whole seedlings (fresh weight 80 mg) from 9-d-old *Col-0* and *SnRK1*^{T175A}, *SnRK1*^{T175D}, and *SnRK1*^{K48M} treated with different durations of submergence were collected, and ground with liquid nitrogen. Subsequently, each sample was re-suspended with 200 µl 2.3% TCA with 40 µl/ml ribitol and centrifuged at 4°C for 15 min at the highest speed. The supernatants were recovered and neutralized to pH 6.5-7 with a few drops of 1M KOH, and then put in an ice bath for 30 min. After 30 min in the ice bath, the samples were centrifuged for 15 min at the highest speed at 4°C, and then the supernatants were collected for LC mass spectrometer quantification. The LC system used for analysis was the ultra-performance liquid chromatography (UPLC) system (ACQUITY UPLC, Waters, Millford, MA). The sample was separated with an ACQUITY UPLC BEH Amide column (particle size 1.7 µm, 1.0 × 150 mm, Waters, Milford, MA) at 80 µl/min flow rate using 13 min gradient for analysis. Fifty millimeters of ammonium acetate in H_2O was used as mobile phase A and ACN was used as mobile phase B. The UPLC system was coupled online to the TSQ Quantum Access Max triple quadrupole mass spectrometer (Thermo Fisher Scientific, San Jose, CA, USA). The instrument was operated in multiple reaction-monitoring (MRM) mode. The signals of AMP and ATP were detected in the negative ion mode. Ribitol was used as the internal standard. The chromatogram acquisition, detection of mass spectral peaks, and their waveform processing were performed using Thermo Xcalibur 2.1 SP1 software (Thermo Scientific, USA).

Antibody and western blot analysis

For western blot examination, protein extraction was performed according to the procedures described in (Hsieh et al., 2012). The western blot experiments were analyzed by anti-phospho-AMPKα (titer 1:1000) (Cell Signaling Technology), anti-p44/P42-MAPK (titer 1:1000) (Cell Signaling Tech.), anti-tubulin (titer 1:5000) (Sigma), anti-cMYC (titer 1:3000) (9E10, The

developmental Studies Hybridoma Bank, University of Iowa), anti-S tag (titer 1:3000) (Novagen), anti-MPK6 (titer 1:3000) (Sigma), anti-GFP (titer 1:3000) (Roche), and SnRK1.1 antibody (titer 1: 2000). SnRK1.1 antibody was raised by a specific peptide with amino acid sequences RASSGYLGAEFQETM.

Protein extraction and in-solution trypsin digestion

Protein extraction was performed according to (Lan et al., 2012). Total proteins were extracted from 9-d-old whole seedlings of *Col-0* and *SnRK1.1*^{K48M} treated with 0 h, 0.5 h, 1 h and 3 h submergence. For general quantitative proteome analysis, 100 mg protein from each sample was used to perform the in solution trypsin digestion. For quantitative phosphoproteome analysis, 3 mg protein of each sample was used to perform the in solution trypsin digestion. The process of the in solution trypsin digestion was as followed: The protein was reduced and alkylated by using dithiothreitol (DTT) and iodoacetamide (IAA), and the DNA and RNA in the sample were degraded by mixing with benzonase nuclease (Novagen) at room temperature for 2 h. Subsequently, endoproteinase Lys-C (Wako) was added and the mixture was incubated at room temperature for 4 h. The peptide solution was further incubated with modified trypsin (Promega) at room temperature overnight. The resulting peptide solution was acidified with 10% trifluoroacetic acid and desalted on Sep-Pak C18 cartridge (Waters).

TiO_2 affinity chromatography and iTRAQ labeling

Phosphopeptides were enriched from each of the digested peptides using TiO2 affinity chromatography according to the instruction manual for the Titansphere Phos-TiO kit (GL Sciences). Eluted peptides were desalted and lyophilized in a centrifugal speed vacuum concentrator. Subsequently, desalted peptides were chemically labeled with iTRAQ reagents (Applied Biosystems) according to the manufacturer's instructions. Samples of *Col-0* that underwent different durations of submergence (0 h, 0.5 h, 1 h, and 3 h) were labeled 113, 114, 115, or 116, respectively, and samples from *SnRK1.1*$^{K48M-5}$ that underwent different durations of submergence were

labeled 117, 118, 119, or 121, respectively. To reduce the peptide complexity of the 8 labeled samples, after labeling, each reaction mixture was aliquoted into two parts in equal volume and these aliquots were mixed into 3 fractions as follows: Fraction 1: 113,114,115 and 116; Fraction 2:117,118,119 and 121; Fraction 3: 113,114,117,and 118. The profile of each fraction in different biological repeats is listed in Table S1.

Strong cation-exchange chromatography fractionation of total proteome quantitative analysis

For total proteome quantitative analysis, the desalted peptide samples from *Col-0* with different treatments were labeled 113-116, and samples from *SnRK1.1*$^{K48M\text{-}5}$ with different treatments were labeled with reagent 117-121, respectively. The labeling reaction was processed at room temperature for 1 h. Subsequently, the labeled samples were combined and fractionated offline using strong cation-exchange chromatography (SCX, Polysulfoethyl A, 5 μm, 200-Å bead, 4.6 × 100 mm) on HPLC. Fractions were collected and pooled into 22 final fractions according to the peak area. Each final fraction was desalted and lyophilized in a centrifugal speed vacuum concentrator. Samples were stored at -80°C before reconstitution in solution for MS analysis.

LC-MS/MS analysis

The SCX fractions were analyzed on a nanoUPLC system (nanoAcquity, Waters) connected to an LTQ-Orbitrap Elite hybrid mass spectrometer (Thermo Scientific). For phosphopeptide quantification, peptide mixtures were loaded onto a 75-μm × 250-mm length BEH130 column (Waters) with C18 resin and eluted using a segmented gradient in 2 h from 5% to 40% solvent B (95% acetonitrile with 0.1% formic acid). Solvent A was 0.1% formic acid in water. The effluent from the C18 column was directly electrosprayed into the mass spectrometer. The LTQ Orbitrap Elite hybrid mass spectrometer instrument was operated in the positive ionization mode. The MS survey scan was performed in the Orbitrap cell, recording a window between 350 and

1,600 m/z. The minimum MS signal for triggering MS/MS was set to 2000. The m/z values triggering MS/MS were put on an exclusion list for 90 s. In all cases, one microscan was recorded. The CID-HCD MS/MS acquisition of the top 10 precursor ions from the survey scan consisted of two sets of independent MS/MS scans, with a CID and a HCD MS/MS experiment triggered from the same precursor ion. CID was conducted with a target value of 10,000 in the linear ion trap with multistage activation enabled, collision energy of 35%, Q value of 0.25 and activation time of 10 ms. HCD-generated ions were detected in the Orbitrap using a target value of 50,000, collision energy of 40% and activation time of 0.1 ms. For total protein quantification, peptide mixtures were separated on C18 BEH130 column and electrosprayed into the mass spectrometer under the same conditions as mentioned above. Full scan MS spectra (m/z 350–1600) were also acquired in the Orbitrap analyzer with a target value of 500,000. The 15 most intense peptide ions with charge states ≥ 2 were sequentially isolated to a target value of 50,000 and fragmented in the HCD cell with normalized collision energy of 35%. The resulting fragment ions were scanned in the Orbitrap analyzer. The ion selection threshold was 5,000 counts for MS/MS, and the maximum allowed ion accumulation times were 10 ms for full scans and 100 ms for HCD-MS/MS. Three independent biological experiments with two technical repeats each were performed.

Phosphorylation motif analysis using motif X

To identify the phosphorylation motifs that were significantly enriched under submergence, all the identified phosphorylated sites of peptides, for which the pRS site probabilities were above 75%, were collected. The phosphorylation sites of the low pRS site probabilities that were caused by closely-spaced multiple phosphorylation sites in the peptide were also selected for motif analysis. Parameters were set to 13 amino acids of peptide length, an occurrence of 10, and a P value $< 10^{-5}$. The default *Arabidopsis* proteomics data set was used as the background data set (which has the same motif patterns as used in the protein sequences of the whole genome

Arabidopsis database TAIR 8 in the Fasta format), as described by (Reiland et al., 2009).

Recombinant protein expression and in vitro kinase assay

The coding sequences of F2KP, PENTA, eIFiso4G1, and PTP1 were cloned into pET32a (Novagen) through Gateway system (Ueguchi-Tanaka et al., 2007) and introduced into *Escherichia coli* Rosseta (DE3) cells. The recombinant S tag-6XHis-fused proteins were purified using Ni-NTA resin (Cube Biotech) following the manufacturer's instructions and examined through S-tag (Novagen) immunoblot. For the *in vitro* kinase, SnRK1.1 was immunoprecipitated from the *Col-0* seedlings that were treated with 1 h submergence or normal conditions. The inactive form of SnRK1.1, SnRK1.1^{K48M}, was immunoprecipitated from *cMYC-SnRK1*^{K48M} transgenic line that was treated with 1 h submergence. Plant material (~1 g) was extracted in 1X volume of IP buffer containing 10 mM DTT, 150 mM NaCl, 50 mM Tris buffer pH 7.6, 10 mM EDTA, 10% NP40, 1/500(v/v) plant-specific protease inhibitor cocktail (Sigma P9599), 1/10 phosphatase inhibitor cocktail (Roche). After centrifugation for 15 min at the highest speed at 4°C, the supernatants were collected, and then 2 mg total protein was incubated with antibody of SnRK1.1 (titer 1:200) overnight at 4°C. After incubation, 30 µl protein A-agarose (Roche) was further added to the sample and incubated overnight at 4°C. The cMYC-SnRK1.1^{K48M} sample was mixed with cMYC-conjugated agarose (BD) and inoculated overnight at 4°C. The mixture was washed three times with wash buffer (10 mM Tris-HCl, pH 7.6, 100 mM NaCl, NP40 0.5%) and resuspended in a total volume of 50 µl of beads buffer (50 mM Tris-HCl, pH 7.6, 250 mM KCl, 10% glycerol, and 0.1% Tween 20), of which 5 µl was used for each reaction.

Immunoprecipitated SnRK1.1 was incubated with recombinant protein (1-0.8 µg of F2KP, PENTA, eIFiso4G1, or PTP1) in 20 µl of kinase buffer (20 mM Tris-Hcl, pH 7.6, 20 mM $MgCl_2$, and 2 mM $MnCl_2$ and 100 µM ATP) for 1 h at 30°C. The kinase reactions were performed with or without λ phosphatase (Biolabs) for 1 h at 30°C. All reaction products were resolved in

a Phos Tag SDS PAGE (50 μM Phos-Tag ligand [Wako], 100 μM ZnCl, 7% acrylamide gel) and S-tag immunoblot, according to the Phos Tag (Wako) protocol. The mobility of phosphorylated protein was selectively retarded by Phos tag.

For mass spectrometer analysis, the *in vitro* kinase product was digested by trypsin (Promega) according to the manufacturer's instructions. Phosphopeptides were then enriched using TiO_2 (Phos-TiO Titansphere, GL Sciences) self-packed in a 200 ml pipette tip. The enriched phosphopeptides were acidified with 5% formic acid, dried in a vacuum, and re-dissolved in 0.1% formic acid for LC-MS analysis. LC LTQ-Orbitrap XL hybrid mass spectrometry was used. LC-MS/MS analysis was performed on a nanoAcquity system (Waters) connected to an LTQ-Orbitrap XL hybrid mass spectrometer (Thermo Fisher Scientific) equipped with a PicoView nanospray interface (New Objective). Peptide mixtures were separated on a C18 UPLC® column (BEH130 C18, 75 mm x 25 cm, 1.7 mm, Waters) using a linear gradient for 60 min from 5% to 40% solvent B (0.1% formic acid in acetonitrile) at 300 nl/min flow rate. Solvent A was 0.1% formic acid in water. The LTQ-Orbitrap XL hybrid mass spectrometer was operated in positive ionization mode. The MS survey scan was performed in the Orbitrap cell in *m*/*z* 350-1600 range with resolving power at 60,000 and automatic gain control (AGC) of 500,000 ions. The data dependent MS/MS data acquisitions were performed with top 10 intensities of the peptide ions from the previous MS survey scan. CID with multistage activation enabled at 97.97, 48.99, and 32.66 Thompson (Th) was used to generate peptide fragments for MS/MS analysis. The MS/MS was set as follows, minimum signal of 2,000 for triggering MS/MS, dynamic exclusion duration of 90 sec, 1 microscan, AGC of 70,000, NCE of 35%, Q value of 0.25, and an activation time of 30 ms. Peptide and protein identification was performed using the Proteome Discoverer software (ver. 1.4, Thermo Fisher Scientific) with SEQUEST and Mascot (ver. 2.5, Matrix Science) search engines against Arabidopsis protein database (TAIR10). The parameter criteria followed the process of database research we mentioned in the Materials and Methods.

Metabolomics extraction and quantification

Whole seedling tissue (100 mg) from 9-d-old seedlings of *Col-0, SnRK1.1*$^{K48M-5}$*,* and *SnRK1.1*$^{K48M-9}$ treated with different durations of submergence were collected and ground with liquid nitrogen. Each sample was re-suspended with 1 ml 70% methanol with 12 µg/ml ribitol, and then sonicated for 30 min at 4°C. Subsequently, samples were centrifuged at the highest speed and 4°C for 15 min. The supernatant of each sample was collected and vacuum dried. The dried samples were then derivatized by bis(trimethylsilyl)-trifluoroacetamide (BSTFA) containing 1% trimethylchlorosilane (TMCS) and analyzed using a Pegasus 4D GCxGC-TOFMS system (LECO, St. Joseph, MI, USA). The first dimension column was a Resteck RTX-5MS (30 m, 0.25 mm i.d., 0.25 mm d.f.) and the second dimension column was a Resteck RTX-200 (1.6 m, 0.18 mm i.d., 0.20 mm d.f.). Sample injection volume was 1 ml with helium as the carrier gas at a flow rate of 1 mL/min. The GC oven temperature was held at 40°C for 5 minutes and programmed at 300°C and held at 300°C for 8 min. The mass range collected was m/z 50–800. Data processing and GCxGC-TOFMS parameters were performed using LECO ChromaTOF software (version 4.43.3.0). Mass spectra were compared against the NIST, LECO/Fiehn and Wiley Registry 9th Edition mass spectrometry libraries.

For trehalose-6-phosphate (T6P) quantification, the process was performed according to (Torano et al. 2012). Whole seedling tissues (120 mg) from 9-d-old *Col-0, SnRK1.1*$^{K48M-5}$ and *SnRK1.1*$^{K48M-9}$ treated with different durations of submergence were collected and ground with liquid nitrogen. Each sample was re-suspended with 500 ml chloroform/ACN (1:2) and 12 µg/ml ^{13}C-Glucose. ^{13}C-Glucose was used as an internal standard. The mixture was sonicated for 30 min at 4°C and the sugars were extracted twice from the organic phase by liquid-liquid extraction with 500 ml water. Subsequently, the aqueous phase was collected and vacuum dried. The dried sample was suspended with a freshly prepared 40 µl of water/MeOH/MeCN 8/7/85 (*v/v/v*) solution and injected 10 µl directly into LC system (ultra-performance liquid chromatography (UPLC) system (ACQUITY UPLC, Waters, Milford, MA, USA)) The sample was separated with ACQUITY UPLC BEH

Amide column (particle size 1.7 μm, 1.0 × 150 mm) at 80 μl/min flow rate using 10 min gradient from 10% to 50% mobile phase A (0.8% Piperidine) in mobile phase B consisting of ACN and 0.8% Piperidine. The UPLC system was coupled online to the Waters Xevo TQ-S triple quadruple mass spectrometer operated in the negative multiple reaction-monitoring (MRM) mode. The collision energy was 10-35 eV. Data acquisition and processing were performed using MassLynx version 4.1 and TargetLynx software (Waters Corp.).

Quantitative polymerase chain reaction

RNA extraction, reverse transcription and qRT-PCR were performed as previously described (Hsu et al., 2013). Sequences of primers are listed in Table S8. TUB3 (AT5G62700) was used as an internal control for normalization. Relative expression levels were calculated by comparing the expression of the gene at a certain time point to the sample of time zero.

Accession numbers of sequences

Sequence data from this article can be found in the *Arabidopsis* Genome initiative or GeneBank/EMBL databases under the following accession numbers: SnRK1.1 (At3g01090.1), SnRK1.2 (At3g29160), Akin β1 (At5g2117), Akin β2 (At4g16360), Akin β3 (At3g48530), Akin γ (At3g48530), Akin βγ (At1g09020), GRIK1 (At5g60550), GRIK2 (At3g45240), PTP1 (At1g71860), MPK3 (AT3g45640), MPK6 (At2g43790), HsfA2 (At2g26150), ADH1 (At1g77120), PIP2 (At2g37170, At4g35100), TPS8 (At1g70290), TPS7 (At1g06410), OZF1 (At2g19810), eIFiso4G-1 (At5g57870), F2KP(At1g07110), ,GRP8(At4g39260), ASK1(At1g51140), ASK2(At1g05805), TOR (At1g50030), S6K1 (At3g08730), ZAT10(At1g27730), PENTA (At3g02830), NHX1 (At5g27150), PORA (At5g54190). PTP1 of Brassica oleracea (XP_013587820.1), PTP1 of Brassica napus (XP_013649509.1), PTP1 of Brassica rapa (XP_009105847.1), PTP1 of Glycine max (NP_001237920.1), PTP1 of Nicotiana tomentosiformis (XP_009609766.1)

References

Hsieh WP, Hsieh HL Wu SH. 2012. *Arabidopsis* bZIP16 transcription factor integrates light and hormone signaling pathways to regulate early seedling development. Plant Cell **24:** 3997-4011.

Lan P, Li W, Wen TN, Schmidt W. 2012. Quantitative phosphoproteome profiling of iron-deficient *Arabidopsis* roots. Plant Physiology **159:** 403-417.

Reiland S, Messerli G, Baerenfaller K, Gerrits B, Endler A, Grossmann J, Gruissem W, Baginsky S. 2009. Large-scale *Arabidopsis* phosphoproteome profiling reveals novel chloroplast kinase substrates and phosphorylation networks. Plant Physiology **150:** 889-903.

Torano JS, Delatte TL, Schluepmann H, Smeekens SCM, Jong GJD, Somsen GW. 2012. Determination of trhalose-6-phosphate in Arabidopsis thaliana seedlings by hydrophilic interaction liquid chromatography mass spectrometery. Analytical and Bioanalytical Chemistry **403**:1353-1360

Ueguchi-Tanaka M, Nakajima M, Katoh E, Ohmiya H, Asano K, Saji S, Hongyu X, Ashikari M, Kitano H, Yamaguchi I, Matsuoka M. 2007. Molecular Interactions of a Soluble Gibberellin Receptor, GID1, with a Rice DELLA Protein, SLR1, and Gibberellin. Plant Cell **19:** 2140-2155.

Supplemental Table 3. Phosphorylation peptides upregulated in Col-0 under submergence. *, indicates phosphorylation site.

			0.5h (114/113)			1h (115/113)			3h (116/113)			
Phospho peptide	AGI number	Description	Mean.Obs	SE.Obs	p<0.05	Mean.Obs	SE.Obs	p<0.05	Mean.Obs	SE.Obs	p<0.05	pRS Site Probabilities
**Cellular carbohydrate metabolic process**												
S*Y*T*NLLDLASGNFPVMGR	AT1G06410.1	TPS7	1.09	0.14	N	1.43	0.25	N	1.32	0.06	Y	S(1): 33.3; Y(2): 33.3; T(3): 33.3; S(10): 0.0
VLS*LS*PS*FRK	AT1G70290.1	TPS8	1.29	0.04	Y	1.47	0.15	Y	1.36	0.05	Y	S(3): 33.3; S(5): 33.3; S(7): 33.3
SVET*LSPFQQK	AT1G07110.1	Fructose-2,6-bisphosphate 2-phosphatase	2.01	0.47	Y	1.49	0.44	N	1.14	0.24	N	S(1): 0.0; T(4): 0.0; S(6): 100.0
LGDGAAES*LHVK	AT3G52930.1	FBA8	1.32	0.08	Y	1.56	0.23	N	2.07	0.20	Y	S(8): 100.0
LGEGAAES*LHVK	AT2G36460.1	FBA7	1.23	0.02	Y	1.22	0.12	N	2.13	0.39	Y	S(8): 100.0
HVES*MS*QLPSGAGK	AT5G36880.2	Plastidic acetyl-coA synthetase	1.41	0.12	Y	1.16	0.26	N	1.17	0.17	N	S(4): 50.0; S(6): 50.0; S(10): 0.0
**Response to stress**												
GFVPFVPGS*PT*ER	AT5G27150.1	NHX1	1.18	0.47	N	1.48	0.16	Y	1.08	0.37	N	S(9): 95.4; T(11): 4.6
MYYQAPS*EFGFGK	AT2G41430.1	Hydrophilic protein lacking Cys residues	0.99	0.04	N	1.31	0.14	N	1.75	0.17	Y	Y(2): 0.5; Y(3): 0.5; S(7): 99.1
GWFGGGATDEVKPES*PHSVEEAPK	AT5G52310.1	RD29A	1.58	0.19	Y	1.33	0.21	N	1.02	0.18	N	T(8): 0.0; S(15): 50.0; S(18): 50.0
EGGGYGGGDGGS*YGGGGGGW	AT4G39260.1	GRP8	0.91	0.14	N	1.00	0.15	N	1.37	0.11	Y	Y(5): 0.0; S(12): 92.4; Y(13): 7.6
DLSVGGPS*HS*LDAENEREVNR	AT1G17210.1	IAP-like protein 1	1.61	0.21	Y	1.22	0.23	N	1.03	0.10	N	S(3): 0.4; S(8): 49.8; S(10): 49.8
T*S*S*AANLFTGSTR	AT1G71860.1	PTP1	1.23	0.10	N	1.43	0.14	Y	1.32	0.03	Y	T(1): 0.0; S(2): 0.6; S(3): 99.4; T(9): 0.0; S(11): 0.0; T(12): 0.0
**Protein metabolism**												
DLS*DFSPK	AT2G23140.1	/U-box superfamily protein with ARM repeat do	0.86	0.06	N	1.37	0.31	N	1.39	0.19	Y	S(3): 0.0; S(6): 100.0
HPWLTYPYEPIS*A	AT3G17750.1	Protein serine/threonine kinase activity	1.33	0.23	N	1.36	0.16	Y	1.06	0.07	N	T(5): 0.0; Y(6): 0.0; Y(8): 0.0; S(12): 100.0
DGPGPLHS*PAVSK	AT5G57870.1	eIFiso4G1	2.15	0.90	Y	1.44	0.04	Y	1.33	0.05	Y	S(8): 100.0; S(12): 0.0
DGEVS*DVEEDEYEAK	AT5G25780.1	eIF3B1	1.37	0.13	Y	0.91	0.23	N	0.77	0.12	N	S(5): 100.0; Y(12): 0.0
**Gene expression**												
S*LGS*NLGNDVVASLR	AT2G19810.1	OZF1	1.27	0.13	N	1.58	0.13	Y	1.78	0.37	N	S(1): 87.4; S(4): 12.6; S(13): 0.0
NSVVS*PVVGAGGDSSK	AT2G37340.1	RS-containing Zinc knuckle protein	1.37	0.07	Y	1.20	0.06	Y	0.91	0.02	Y	S(2): 9.8; S(5): 90.2; S(14): 0.0; S(15): 0.0
ASFIAS*PR	AT3G02830.1	PENTA 1	1.28	0.07	Y	1.53	0.02	Y	0.87	0.16	N	T(3): 0.0; Y(4): 0.0; T(7): 0.0; S(9): 49.8; T(11): 49.8; T(17): 0.2; S(18): 0.2; T(19): 0.2
IKS*PSATTAAAPPAK	AT1G61730.1	ding storekeeper protein-related transcriptional	1.33	0.02	Y	1.10	0.17	N	0.93	0.18	N	S(3): 92.1; S(5): 7.1; T(7): 0.7; T(8): 0.1
ELAEQIAKDPS*FNQLAEQLQR	AT2G17390.1	AKR2A	1.43	0.22	N	1.51	0.08	Y	1.72	0.24	Y	S(11): 100.0
**Transporter**												
SLGS*FRS*AANV	AT2G37170.1	PIP2	1.38	0.16	N	1.83	0.15	Y	1.89	0.19	Y	S(1): 0.0; S(4): 100.0; S(7): 100.0
DSSSIAGSS*S*PIPATVSTGK	AT2G38410.1	ENTH/VHS/GAT family protein	1.48	0.13	Y	1.29	0.24	N	1.02	0.12	N	S(2): 0.0; S(3): 0.0; S(4): 0.0; S(8): 0.2; S(9): 49.7; S(10): 49.7; T(15): 0.2; S(17): 0.2; T(18): 0.0
SLNASFAAT*S*ANS*AS*RS*NIVDWAEK	AT2G17980.1	SLY1 gene family	1.34	0.01	Y	0.98	0.14	N	1.10	0.16	N	S(1): 0.0; S(5): 0.0; T(9): 20.0; S(10): 20.0; S(13): 20.0; S(15): 20.0; S(17): 20.0
ALGS*FRSNAT*N	AT4G35100.1	PIP3	1.04	0.19	N	1.34	0.32	N	1.55	0.13	Y	S(4): 100.0; S(7): 100.0; T(10): 0.0
NIEDIFS*S*GS*RR	AT1G59870.1	ATP binding cassette transporter	1.66	0.37	N	1.60	0.23	Y	1.08	0.09	N	S(7): 33.3; S(8): 33.3; S(10): 33.3
**Nitrogen compound metabolic process**												
DGQIEETVDT*APASLGSPSR	AT5G67220.1	FMN-linked oxidoreductases superfamily protei	2.01	0.21	Y	1.09	0.11	N	1.17	0.12	N	T(7): 0.7; T(10): 98.0; S(14): 0.7; S(17): 0.7; S(19): 0.0
**Other function**												
EAPVGFTPPQLDPNTPSPIFAGS*T*GGLLR	AT1G03130.1	Photosysten subunit D2	1.05	0.34	N	1.07	0.19	N	1.37	0.00	Y	T(7): 0.0; T(15): 0.1; S(17): 10.9; S(23): 78.2; T(24): 10.9
ASS*GPDLSEIPNPADFPVIRK	AT5G59210.1	myosin heavy chain-related	1.29	0.14	N	1.22	0.22	N	1.38	0.07	Y	S(2): 0.7; S(3): 98.5; S(8): 0.7
SEEQEEGS*Y*RKPSYGR	AT5G39570.1	Unknown	1.71	0.18	Y	1.80	0.08	Y	0.68	0.27	N	S(1): 0.0; S(8): 49.7; Y(9): 49.7; S(13): 0.3; Y(14): 0.3
TSS*LLSSSVASDTLR	AT5G35430.1	atricopeptide repeat (TPR)-like superfamily pro	1.52	0.17	Y	1.57	0.12	Y	1.20	0.17	N	T(1): 0.2; S(2): 0.2; S(3): 99.5; S(6): 0.0; S(7): 0.0; S(8): 0.0; S(11): 0.0; T(13): 0.0
SFS*VADFPR	AT5G21940.1	Unknown	1.50	0.07	Y	1.82	0.03	Y	1.49	0.25	N	S(1): 0.5; S(3): 99.5
SFT*NLT*AEAASALTSSSSMK	AT5G21940.1	Unknown	1.09	0.13	N	1.51	0.39	N	1.94	0.14	Y	S(1): 33.3; T(3): 33.3; T(6): 33.3; S(11): 0.2; T(14): 0.0; S(15): 0.0; S(16): 0.0; S(17): 0.0; S(18): 0.0
TVQFVDWCPT*GFK	AT5G19770.1	TUBULIN ALPHA-3	1.12	0.03	Y	1.49	0.10	Y	1.63	0.07	Y	T(2): 0.0; T(11): 100.0
SYS*GSLYR	AT5G14720.1	Protein kinase superfamily protein	1.30	0.00	Y	1.23	0.21	N	1.25	0.11	Y	S(1): 0.0; Y(2): 0.6; S(3): 99.4; S(5): 0.0; Y(7): 0.0
LFLFPSSPISGGFGS*EGSTK	AT5G09620.1	Octicosapeptide/Phox/Bem1p family protein	1.32	0.00	Y	0.96	0.02	N	1.51	0.11	Y	S(6): 0.0; S(7): 0.0; S(10): 0.0; S(15): 100.0; S(18): 0.0; T(19): 0.0
SLIEDEAELS*DWVSDLR	AT5G08610.1	ning nucleoside triphosphate hydrolases super	1.57	0.03	Y	1.14	0.16	N	1.37	0.15	Y	S(1): 0.2; S(10): 99.6; S(14): 0.2
SGFSLS*T*IER	AT5G08050.1	Unknown	1.23	0.21	N	1.48	0.07	Y	1.02	0.14	N	S(1): 0.0; S(4): 0.2; S(6): 49.9; T(7): 49.9
S*ASSLDLNLR	AT4G32340.1	atricopeptide repeat (TPR)-like superfamily pro	1.12	0.01	Y	1.38	0.05	Y	1.65	0.11	Y	S(1): 99.5; S(3): 0.5; S(4): 0.0
VDVYNRVNS*IPR	AT4G27450.1	minium induced protein with YGL and LRDR mo	1.23	0.28	N	1.32	0.10	Y	2.15	0.35	Y	Y(4): 0.0; S(9): 100.0
VDS*EGVLCGANFK	AT4G27450.1	minium induced protein with YGL and LRDR mo	0.87	0.10	N	1.39	0.26	N	1.56	0.09	Y	S(3): 100.0
SSLNAWGTS*S*LSPR	AT4G24680.1	Modifier of SNC1	1.61	0.28	Y	1.03	0.16	N	0.89	0.03	Y	S(1): 0.0; S(2): 0.0; T(8): 0.2; S(9): 0.2; S(10): 49.8; S(12): 49.8
APEPAAPDNASLLELFEYS*NVNR	AT4G01090.1	Unknown	0.89	0.49	N	1.31	0.01	Y	1.41	0.49	N	S(11): 0.0; Y(18): 6.8; S(19): 93.2
YSLDNNPSSVLS*PR	AT4G00752.1	UBX domain-containing protein	1.61	0.06	Y	1.45	0.05	Y	1.03	0.02	N	Y(1): 0.0; S(2): 0.0; S(8): 0.0; S(9): 0.0; S(12): 100.0
IDS*EGVLCGASFK	AT3G15450.1	minium induced protein with YGL and LRDR mo	1.18	0.14	N	1.95	0.39	N	2.14	0.24	Y	S(3): 100.0; S(11): 0.0
RGS*EANWALANSR	AT3G15450.1	minium induced protein with YGL and LRDR mo	1.10	0.18	N	1.37	0.15	N	1.70	0.11	Y	S(3): 100.0; S(12): 0.0
TYVADVS*EY*LGS*NS*PRDPYLER	AT3G11330.1	Plant Intracellular Ras-group-related LRRs	1.53	0.17	Y	1.68	0.54	N	0.94	0.02	Y	T(1): 0.0; Y(2): 0.0; S(7): 25.0; Y(9): 25.0; S(12): 25.0; S(14): 25.0; Y(19): 0.0
TNS*LLVLPR	AT3G05200.1	RING-H2 zinc finger protein ATL6 (ATL6)	1.27	0.17	N	1.44	0.20	Y	1.13	0.06	N	T(1): 0.4; S(3): 99.6
SIPGAPPVT*PAGSFGRK	AT2G47970.1	Nuclear pore localisation protein NPL4	1.48	0.12	Y	1.17	0.15	N	1.20	0.01	Y	S(1): 0.0; T(9): 99.0; S(13): 1.0
MYYQAPS*EFGFGK	AT2G41430.1	ERD15	0.99	0.04	N	1.31	0.14	N	1.75	0.17	Y	Y(2): 0.5; Y(3): 0.5; S(7): 99.1
S*LLQTFEAK	AT2G30930.1	Unknown	1.15	0.21	N	1.05	0.09	N	1.36	0.09	Y	S(1): 99.9; T(5): 0.1
NEESVLLFPELILS*PQERPPSR	AT2G20960.1	PEARLI4	1.56	0.11	Y	0.93	0.22	N	1.10	0.03	Y	S(4): 0.0; S(14): 100.0; S(21): 0.0
VSS*QFLPPDGSR	AT2G20050.1	Protein serine/threonine phosphatases	1.30	0.02	Y	1.24	0.45	N	1.14	0.08	N	S(2): 0.9; S(3): 99.1; S(11): 0.0
EIITLDPS*S*PKVPAVVPVK	AT1G67950.3	NA-binding (RRM/RBD/RNP motifs) family prote	1.62	0.23	Y	1.29	0.07	Y	1.25	0.24	N	T(4): 0.0; S(8): 50.0; S(9): 50.0
IT*T*QPINIR	AT1G28330.5	Dormancy-associated protein (DRM1)	1.33	0.16	N	1.82	0.21	Y	1.63	0.23	Y	T(2): 5.7; T(3): 94.3
ASLTRFDS*IGSTR	AT1G21630.2	Calcium-binding EF hand family protein	1.55	0.13	Y	1.07	0.11	N	1.03	0.22	N	S(2): 0.0; T(4): 0.0; S(8): 100.0; S(11): 0.0; T(12): 0.0
SQFAFDDSVPS*T*PLSR	AT1G20760.1	Calcium-binding EF hand family protein	1.30	0.04	Y	1.59	0.30	N	1.05	0.01	Y	S(1): 0.0; S(8): 0.0; S(11): 10.9; T(12): 88.8; S(15): 0.2
FGNS*PPRFSDASAR	AT1G20760.1	Calcium-binding EF hand family protein	1.04	0.01	N	1.39	0.09	Y	1.38	0.22	N	S(4): 100.0; S(9): 0.0; S(12): 0.0
SFS*QAEFLEANTVLSK	AT1G15040.1	GAT1_2.1	0.89	0.20	N	1.10	0.21	N	1.68	0.17	Y	S(1): 0.4; S(3): 99.6; T(12): 0.0; S(15): 0.0
ASS*FVYSPGR	AT1G08680.4	AGD14	1.54	0.60	N	1.36	0.13	Y	1.29	0.11	Y	S(2): 0.8; S(3): 99.2; Y(6): 0.0; S(7): 0.0
LT*S*LS*AS*S*PVEIILDK	AT1G07990.1	SIT4 phosphatase-associated family protein	1.57	0.32	N	1.38	0.12	Y	1.26	0.03	Y	T(2): 20.0; S(3): 20.0; S(5): 20.0; S(7): 20.0; S(8): 20.0
TRDSDEEDRDY*DVAALANNLSQAFR	AT1G07990.1	SIT4 phosphatase-associated family protein	1.07	0.29	N	1.73	0.72	N	1.40	0.04	Y	T(1): 0.0; S(4): 0.0; Y(11): 100.0; S(21): 0.0
TIQFVDWCPT*GFK	AT1G04820.1	Alpha tubulin	1.11	0.08	N	1.62	0.17	Y	1.56	0.12	Y	T(2): 0.0; T(11): 100.0

Footnote: Asterisk indicted the phosphorylation site.
The nubmers indicate the average value and standard deviation of phosphorylation fold change of all biological repeats.
The time point data that is significantly different (P < 0.05) is marked as “Y”. If not, marked as "N"

Supplemental Table 4. Phosphorylation peptides downregulated in Col-0 under submergence. *, indicates phosphorylation site.

Phospho peptide	AGI number	Description	0.5h (114/113)			1h (115/113)			3h (116/113)			
			Mean.Obs	SE.Obs	p<0.05	Mean.Obs	SE.Obs	p<0.05	Mean.Obs	SE.Obs	p<0.05	pRS Site Probabilities
**Cellular carbohydrate metabolic process**												
EQGRNDAEEDLLS*ELS*EGEK	AT4G10120.1	SPS4F	0.84	0.12	N	0.74	0.01	Y	0.61	0.03	Y	S(13): 50.0; S(16): 50.0
**Protein metabolism**												
EES*EEEEGDFGFDLFG	AT4G25890.1	60S acidic ribosomal protein family	0.87	0.10	N	0.27	0.07	Y	0.88	0.03	Y	S(3): 100.0
ASANSLS*APIK	AT4G10730.1	Protein kinase superfamily protein	0.83	0.31	N	0.55	0.16	Y	0.53	0.01	Y	S(2): 0.0; S(5): 0.0; S(7): 100.0
VEEKEES*DEEDY*GGDFGLFDEE	AT3G09200.1	Ribosomal protein L10 family protein	0.54	0.15	Y	0.41	0.10	Y	0.84	0.43	N	S(7): 50.0; Y(12): 50.0
EES*DDDMGFSLFE	AT2G27710.1	60S acidic ribosomal protein family	0.66	0.10	Y	0.76	0.19	N	0.89	0.02	Y	S(3): 100.0; S(10): 0.0
**Transporters**												
TLS*S*T*PLALVGAK	AT5G40890.1	Voltage-dependent chloride channel.	1.04	0.23	N	1.02	0.24	N	0.59	0.07	Y	T(1): 0.1; S(3): 33.3; S(4): 33.3; T(5): 33.3
EILQSES*FKEEGYLASELQEAEK	AT1G22530.1	PATL2	0.82	0.13	N	0.71	0.09	Y	0.62	0.05	Y	S(5): 1.0; S(7): 97.8; Y(13): 1.0; S(16): 0.1
**Gene expression**												
SLS*DIEQLLSDSIPCK	AT1G51140.1	AKS1	0.50	0.03	Y	0.45	0.07	Y	0.44	0.07	Y	S(1): 14.3; S(3): 85.0; S(10): 0.6; S(12): 0.1
QRS*SPADFFTYLASDK	AT1G05805.1	AKS2	0.77	0.05	Y	0.93	0.19	N	0.55	0.13	Y	S(3): 92.8; S(4): 7.2; T(10): 0.0; Y(11): 0.0; S(14): 0.0
SS*PADFFTYLASDK	AT1G05805.1	AKS2	0.65	0.07	Y	1.19	0.23	N	0.66	0.11	Y	S(1): 0.8; S(2): 99.2; T(8): 0.0; Y(9): 0.0; S(12): 0.0
YGSAPGS*FLNSVVDEVIGGGSSNAR	AT1G05805.1	AKS2	0.53	0.03	Y	0.71	0.10	Y	0.83	0.26	N	Y(1): 1.0; S(3): 8.5; S(7): 82.1; S(11): 8.5; S(21): 0.0; S(22): 0.0
**Nitrogen compound metabolic process**												
EALPALTDELIGDMMS*PPWR	AT3G23490.1	Cyanase	0.53	0.15	Y	0.86	0.30	N	0.94	0.21	N	T(7): 0.0; S(16): 100.0
**Other function**												
AAAS*KT*IEVEVDKPLGLTLGQK	AT5G17170.1	SOS3-1	0.62	0.23	N	0.48	0.19	Y	0.52	0.04	Y	S(4): 50.0; T(6): 50.0; T(18): 0.0
NLAGDVIGT*RT*EAADAK	AT5G01530.1	Light harvesting complex photosystem II	0.71	0.22	N	0.61	0.24	N	0.53	0.17	Y	T(9): 50.0; T(11): 50.0
S*VLS*AFFVK	AT2G46225.2	ABIL1	0.69	0.07	Y	0.76	0.16	N	0.54	0.07	Y	S(1): 50.0; S(4): 50.0
VLSPY*SLPASLLHSER	AT2G46630.1	Unknown	0.55	0.11	Y	0.61	0.12	Y	0.56	0.13	Y	S(3): 9.6; Y(5): 80.7; S(6): 9.6; S(10): 0.0; S(14): 0.0
IDLDKPEVEDDDDNDEDDS*EDDDEAEGHDGEAGGR	AT5G13850.1	NACA3	0.67	0.21	N	0.72	0.38	N	0.57	0.19	Y	S(19): 100.0
AVDSLPLPRPSS*SEVR	AT3G07660.1	e-related protein of unknown function (DUF	0.90	0.22	N	0.65	0.09	Y	0.57	0.05	Y	S(4): 0.7; S(11): 7.3; S(12): 84.6; S(13): 7.3
DT*S*GDY*EDMLVALLGHGDA	AT5G65020.1	ANNAT2	1.16	0.12	N	0.60	0.20	N	0.63	0.12	Y	T(2): 15.9; S(3): 15.9; Y(6): 68.1
FENVFSSISSS*PTK	AT4G12770.1	DnaJ-domain superfamily protein	1.04	0.21	N	0.68	0.23	N	0.64	0.08	Y	S(6): 0.0; S(7): 0.0; S(9): 0.0; S(10): 4.5; S(11): 95.5; T(13): 0.0
NSFFSPPT*PSR	AT5G03040.1	IQD2	0.97	0.22	N	0.77	0.32	N	0.64	0.05	Y	S(2): 0.0; S(5): 0.6; T(8): 98.9; S(10): 0.6
VEEIRS*PQTINK	AT4G01290.1	Unknown	1.40	0.25	N	1.24	0.35	N	0.64	0.11	Y	S(6): 99.9; T(9): 0.1
DSEIEETEEFDTES*PLPK	AT5G55210.1	Unknown	1.17	0.20	N	0.71	0.10	Y	0.66	0.12	Y	S(2): 0.0; T(7): 0.0; T(12): 0.8; S(14): 99.2
APSPVNNPLLGS*LPK	AT1G80490.2	TOPLESS-related 1 (TPR1)	0.88	0.19	N	0.85	0.14	N	0.68	0.12	Y	S(9): 100.0; S(18): 0.0
VQVAT*VRGQAK	AT3G46780.1	Plastid transcriptionally active 16 (PTAC16	1.31	0.53	N	0.98	0.35	N	0.69	0.02	Y	T(5): 100.0
DNDGNES*PRKLDLDGLTPFEK	AT1G55150.1	DEA(D/H)-box RNA helicase family protein	0.99	0.09	N	0.54	0.07	Y	0.74	0.19	N	S(7): 99.1; T(17): 0.9
ALPPDVLFIADPEGSLLGT*GNSVGPTFVGNETR	AT1G70570.2	anthranilate phosphoribosyltransferase	1.20	0.12	N	0.68	0.05	Y	2.31	1.31	N	S(15): 0.6; T(19): 92.1; S(22): 7.3; T(26): 0.0; T(32): 0.0
MEVDAPS*PIAGSVDLADNIAAK	AT1G58025.2	NA-binding bromodomain-containing prote	0.76	0.03	Y	0.69	0.00	Y	0.80	0.06	Y	S(7): 99.9; S(12): 0.1
SSPGGRS*PGFETGSR	AT4G13350.1	GTPase	0.65	0.09	Y	0.74	0.05	Y	0.87	0.07	N	S(1): 0.0; S(2): 0.0; S(7): 100.0; T(12): 0.0; S(14): 0.0

Footnote: Asterisk indicted the phosphorylation site.
The nubmers indicate the average value and standard deviation of phosphorylation fold change of all biological repeats.
The time point data that is significantly different (P < 0.05) is marked as “Y”. If not, marked as "N"

Supplemental Table 5. Differential phosphorylation of selected submergence- responsive genes in *SnRK1.1^{K48M}* mutants. *, indicates phosphorylation site.

		Col-0									SnRK1K48M-5												
	Time (h)	0.5			1			3			0			0.5			1			3			
Phospho peptide	AGI number	mean	(±)SD	P<0.05	mean	(±)SD	P<0.05	mean	(±)SD	P<0.05	mean	(±)SD	P<0.05	mean	(±)SD	P<0.05	mean	(±)SD	P<0.05	mean	(±)SD	P<0.05	Note
**Up regulated in Col-0**																							
**Cellular carbohydrate metabolic process**																							
S*Y*T*NLLDLASGNFPVMGR	AT1G06410	1.09	0.14	N	1.43	0.25	N	1.32	0.06	Y	0.76	0.04	Y	0.91	0.19	N	1.00	0.22	N	1.04	0.14	N	Trehalose-6-phosphatase synthase S7
SVETLS*PFQQK	AT1G07110	2.01	0.47	Y	1.49	0.44	N	1.14	0.03	N	1.52	0.58	N	1.01	0.23	N	1.04	0.14	N	0.91	0.00	Y	Fructose-6-phosphate 2-kinase/fructose-2,6-bisphosphatase
HVES*MS*QLPSGAGK	AT5G36880	1.41	0.12	Y	1.16	0.26	N	1.17	0.17	N	1.33	0.13	N	0.92	0.15	N	0.97	0.17	N	0.94	0.16	N	Plastidic acetyl-coA synthetase
**Response to stress/Gene expression**																							
T*S*S*AANLFTGSTR	AT1G71860	1.23	0.10	N	1.43	0.14	Y	1.32	0.03	Y	NA			0.98	0.07	N	0.89	0.35	N	1.00	0.03	N	PTP1
EGGGYGGGDGGS*Y*GGGGGGW	AT4G39260	0.91	0.14	N	1.00	0.15	N	1.37	0.11	Y	1.06	0.10	N	0.84	0.04	Y	0.72	0.02	Y	0.98	0.07	N	GRP8
ASFIAS*PR	AT3G02830	1.28	0.07	Y	1.53	0.02	Y	1.18	0.07	Y	1.35	0.14	N	1.10	0.08	N	NA			NA			PENTA 1
DGPGPLHS*PAVSK	AT5G57870	2.15	0.90	Y	1.44	0.04	Y	1.33	0.05	Y	1.05	0.06	N	1.37	0.43	N	NA			NA			Eukaryotic translation initiation factor isoform 4G1
**Osmotic regulation**																							
SLGS*FRS*AANV	AT2G37170	1.38	0.16	N	1.83	0.15	Y	1.89	0.19	Y	1.21	0.22	N	0.84	0.11	N	0.82	0.14	N	0.99	0.14	N	PIP2
ALGS*FRS*NATN	AT4G35100	1.04	0.19	N	1.34	0.32	N	1.54	0.13	Y	1.21	0.07	Y	0.83	0.07	N	1.00	0.13	N	1.37	0.23	N	PIP2
GFVPFVPGS*PTER	AT5G27150	1.18	0.47	N	1.48	0.16	Y	1.08	0.37	N	NA			0.88	0.15	N	0.92	0.07	N	1.08	0.10	N	Vacuolar sodium/proton antiporter
**Calcium signaling**																							
ASLTRFDS*IGSTR	AT1G21630	1.55	0.13	Y	1.07	0.07	N	1.03	0.15	N	1.09	0.15	N	1.12	0.09	N	0.96	0.08	N	0.97	0.13	N	Calcium binding EF hand family protein
**Kinase and phosphatase**																							
HPWLTYPYEPIS*A	AT3G17750	1.33	0.23	N	1.36	0.16	Y	1.06	0.07	N	1.08	0.05	N	0.80	0.09	Y	0.90	0.11	N	0.81	0.06	Y	Protein kinase
**Unknown protein**																							
APEPAAPDNASLLELFEY*S*NVNR	AT4G01090	0.89	0.49	N	1.31	0.01	Y	1.41	0.49	N	3.15	2.50	N	0.86	0.06	N	1.22	0.21	N	1.29	0.35	N	Unknown protein
SEEQEEGS*Y*RKPSYGR	AT5G39570	1.71	0.18	Y	1.80	0.08	Y	0.68	0.27	N	NA			1.25	0.07	Y	1.28	0.23	N	1.70	0.45	N	Unknown protein
S*LLQTFEAK	AT2G30930	1.15	0.21	N	1.05	0.09	N	1.36	0.09	Y	NA			1.22	0.12	N	1.15	0.08	N	1.05	0.05	N	Unknown protein
SFS*VADFPR	AT5G21940	1.50	0.07	Y	1.82	0.03	Y	1.49	0.25	Y	NA			1.23	0.04	Y	1.28	0.21	N	1.51	0.20	N	Unknown protein
**Other function**																							
S*ASSLDLNLR	AT4G32340	1.1207	0.0138	Y	1.3789	0.0473	Y	1.651	0.1063	Y	1.1918	0.0065	Y	1.1715	0.2297	N	1.1696	0.0702	Y	1.2852	0.0962	Y	at (TPR)-like superfamily protein
ASS*FVYSPGR	AT1G08680	1.54	0.60	N	1.36	0.13	Y	1.29	0.11	Y	1.10	0.18	N	1.20	0.17	N	1.09	0.30	N	0.97	0.10	N	ARF GTPase 14
NEESVLLFPELILS*PQERPPSR	AT2G20960	1.56	0.11	Y	0.93	0.34	N	1.10	0.03	N	0.98	0.13	N	0.84	0.15	N	0.75	0.18	N	1.01	0.05	N	PEARLI4
TYVADVSEY*LGS*NS*PRDPYLER	AT3G11330	1.53	0.17	Y	1.68	0.54	N	0.94	0.02	Y	1.11	0.17	N	1.02	0.12	N	0.95	0.10	N	0.85	0.09	Y	Plant intracellular ras group-related LRR 9
TRDSDEEDRDY*DVAALANNLSQAFR	AT1G07990	1.07	1.07	N	1.73	0.72	N	1.40	0.04	Y	0.61	0.28	N	0.93	0.07	N	1.04	0.27	N	0.96	0.08	N	SIT4 phosphatase-associated family protein
ITT*QPINIR	AT1G28330	1.33	0.16	N	1.82	0.21	Y	1.63	0.23	Y	1.28	0.24	N	1.04	0.10	N	1.17	0.26	N	1.43	0.26	N	Dormancy-associated protein 1
SLNASFAAT*S*ANS*AS*RS*NIVDWAEK	AT2G17980	1.34	0.01	Y	0.98	0.14	N	1.10	0.16	N	NA			0.98	0.34	N	1.23	0.18	N	1.33	0.20	N	SLY1
NSVVS*PVVGAGGDSSK	AT2G37340	1.37	0.07	Y	1.20	0.06	Y	0.91	0.02	Y	1.48	0.32	N	0.61	0.19	N	0.82	0.09	N	0.70	0.16	N	Arginine/serine-rich zinc knuckle-containing protein 33,
DSSSIAGSS*S*PIPATVSTGK	AT2G38410	1.48	0.19	Y	1.29	0.10	N	1.02	0.05	N	1.16	0.06	Y	0.81	0.24	N	0.71	0.15	N	0.77	0.12	N	ENTH/VHS/GAT family protein
TNS*LLVLPR	AT3G05200	1.27	0.17	N	1.44	0.20	Y	1.13	0.06	N	1.04	0.12	N	1.14	0.38	N	0.82	0.38	N	1.15	0.15	N	Putative RING-H2 zinc finger protein ATL6
SSLNAWGTSS*LS*PR	AT4G24680	1.61	0.28	Y	1.03	0.16	N	0.89	0.03	Y	1.14	0.20	N	1.03	0.13	N	0.87	0.10	N	1.00	0.09	N	Modifier of SNC1
SLIEDEAELS*DWVSDLR	AT5G08610	1.57	0.03	Y	1.14	0.16	N	1.37	0.15	Y	NA			0.91	0.40	N	1.54	0.29	N	2.26	0.78	N	P-loop containing nucleoside triphosphate hydrolases
LFLFPSSPISGGFGS*EGSTK	AT5G09620	1.32	0.00	Y	0.96	0.02	N	1.51	0.11	Y	NA			1.13	0.11	N	17.83	17.05	N	1.17	0.07	N	Octicosapeptide/Phox/Bem1p family protein
AS*S*GPDLS*EIPNPADFPVIRK	AT5G59210	1.29	0.14	N	1.22	0.22	N	1.38	0.07	Y	1.14	0.41	N	1.22	0.36	N	1.32	0.34	N	1.27	0.20	N	Myosin heavy chain-related
DGQIEETVDT*APASLGSPSR	AT5G67220	2.01	0.21	Y	1.09	0.11	N	1.17	0.12	N	1.15	0.36	N	1.12	0.01	Y	1.14	0.10	N	0.96	0.10	N	FMN-linked oxidoreductases superfamily protein
SIPGAPPVT*PAGSFGRK	AT2G47970	1.48	0.12	Y	1.17	0.15	N	1.20	0.01	Y	1.23	0.02	Y	1.01	0.17	N	1.12	0.01	Y	1.30	0.11	Y	Nuclear pore localisation protein NPL4
TSS*LLSSSVASDTLR	AT5G35430	1.52	0.17	Y	1.57	0.12	Y	1.20	0.17	N	0.99	0.07	N	1.05	0.17	N	1.03	0.21	N	1.28	0.01	Y	Tetratricopeptide repeat (TPR)-like superfamily protein
**Down regulated in Col-0**																							
**Protein synthesis**																							
EES*DDMGFSLFE	AT2G27710	0.66	0.10	Y	0.76	0.19	N	0.89	0.02	Y	0.85	0.02	Y	1.33	0.17	N	0.75	0.15	N	1.22	0.08	N	60S acidic ribosomal protein family
**ABA response**																							
SLS*DIEQLLSDSIPCK	AT1G51140	0.50	0.03	Y	0.45	0.07	Y	0.44	0.07	Y	0.68	0.20	N	0.66	0.05	Y	0.79	0.19	N	0.58	0.25	N	AKS1
YGSAPGS*FLNSVVDEVIGGGSSNAR	AT1G05805	0.53	0.03	Y	0.71	0.19	N	0.55	0.13	Y	0.64	0.02	Y	0.86	0.17	Y	0.95	0.14	N	0.85	0.13	N	AKS2
APSPVNNPLLGS*LPK	AT1G80490	0.88	0.19	N	0.85	0.14	N	0.68	0.12	Y	NA			0.81	0.06	Y	0.84	0.07	Y	1.01	0.03	N	TOPLESS-related 1
VLSPY*SLPASLLHSER	AT2G46630	0.55	0.11	Y	0.61	0.12	Y	0.56	0.13	Y	0.72	0.06	Y	0.86	0.12	N	0.95	0.04	N	0.94	0.17	N	Unknown protein
DSEIEETEEFDTES*PLPK	AT5G55210	1.17	0.20	N	0.71	0.10	Y	0.66	0.12	Y	0.77	0.11	N	0.84	0.07	N	0.80	0.16	N	0.81	0.10	N	Unknown protein
**Other funciton**																							
EILQSES*FKEEGYLASELQEAEK	AT1G22530	0.82	0.13	N	0.71	0.09	Y	0.62	0.05	Y	0.74	0.18	N	1.16	0.25	N	1.26	0.08	Y	1.08	0.04	N	Patellin 2
LPPDVLFIADPEGSLLGT*GNSVGPTFVGNETI	AT1G70570	1.20	0.12	N	0.68	0.05	Y	2.31	1.31	N	NA			0.72	0.15	N	1.07	0.10	N	3.49	2.50	N	Anthranilate phosphoribosyltransferase
EALPALTDELIGDMMS*PPWR	AT3G23490	0.53	0.15	Y	0.86	0.30	N	0.94	0.21	N	0.79	0.00	Y	1.00	0.15	N	1.08	0.04	N	1.07	0.02	N	Cyanase
AAAS*KT*IEVEVDKPLGLTLGQK	AT5G17170	0.62	0.23	N	0.48	0.19	Y	0.52	0.04	Y	1.26	0.26	N	1.35	0.34	N	1.33	0.44	N	0.85	0.15	N	Enhancer of sos3-1 (ENH1)
DKPEVEDDDDNDEDDS*EDDDEAEGHDGEAC	AT5G13850	0.67	0.21	N	0.72	0.38	N	0.57	0.19	Y	NA			0.98	0.16	N	0.49	0.29	N	0.74	0.09	Y	Nascent polypeptide-associated complex subunit alpha-like protein 3

The pRS score of these phosphorylated peptides in Col-0 was passed the criteria (>50), but that in SnRK1.1K48M-5 was not.

		Col-0									SnRK1K48M-5												
	Time (h)	0.5			1			3			0			0.5			1			3			
Phospho peptide	AGI number	mean	(±)SD	P<0.05	mean	(±)SD	P<0.05	mean	(±)SD	P<0.05	mean	(±)SD	P<0.05	mean	(±)SD	P<0.05	mean	(±)SD	P<0.05	mean	(±)SD	P<0.05	
**Cellular carbohydrate metabolic process**																							
VLS*LS*PS*FRK	AT1G70290	1.29	0.04	Y	1.47	0.15	Y	1.36	0.05	Y	NA			0.86	0.10	N	0.88	0.14	N	1.12	0.22	N	Trehalose-6-phosphatase synthase S8
**Response to stress/ Gene expression**																							
EAPVGFTPPQLDPNTPS*PIFAGS*T*GGLLR	AT1G03130	1.05	0.34	N	1.07	0.19	N	1.37	0.00	Y	NA			0.97	0.20	N	1.35	0.11	Y	8.98	8.02	N	Photosystem I subunit d-2
DLSVGGPS*HS*LDAENEREVNR	AT1G17210	1.61	0.04	Y	1.22	0.23	N	1.03	0.22	N	NA			0.83	0.10	N	0.88	0.04	Y	0.83	0.03	N	Arabidopsis IAP-like protein
**Unknown protein**																							
SGFSLS*T*IER	AT5G08050	1.23	0.21	N	1.48	0.07	Y	1.02	0.14	N	0.87	0.07	N	0.65	0.12	Y	0.62	0.14	Y	0.90	0.08	Y	Unknown protein

Footnote: Asterisk indicted the phosphorylation site.
The nubmers indicate the average value and standard deviation of phosphorylation fold change of all biological repeats.
The time point data that is significantly different (P < 0.05) is marked as “Y”. If not, marked as "N"
The time point data that is not detected in the three batches or detected once in three batches is marked as NA (not available).

Supplemental Table 6. Protein abundance of differential phosphorylation responsive genes in *SnRK1.1*^{K48M} mutants.

	Col-0						SnRK1K48M-5								
Time (h)	0.5		1		3		0		0.5		1		3		
AGI number	mean	(±)SD	mean	(±)SD	mean	(±)SD	mean	(±)SD	mean	(±)SD	mean	(±)SD	mean	(±)SD	Description
Phosphorylation level Up regulated in Col-0															
AT1G06410.1	1.11	0.25	1.15	0.16	1.34	0.01	1.05	0.14	1.07	0.48	1.27	0.42	1.30	0.25	TPS7
AT1G07110.1	1.16	0.21	1.20	0.44	1.08	0.41	1.13	0.14	0.99	0.11	1.50	1.07	1.33	0.36	ctose-2,6-bisphosphate 2-phosphatase
AT3G52930.1	0.94	0.03	0.90	0.09	0.94	0.04	1.01	0.05	1.15	0.20	0.94	0.08	0.91	0.13	FBA8
AT5G36880.2	0.96	0.14	0.96	0.17	0.90	0.19	0.98	0.14	1.01	0.14	1.08	0.13	1.55	0.63	Plastidic acetyl-coA synthetase
AT1G03130.1	0.81	0.05	1.44	0.89	1.10	0.30	1.13	0.42	0.99	0.32	0.72	0.27	0.86	0.38	Photosysten subunit D2
AT1G17210.1	0.97	0.06	0.92	0.15	0.99	0.03	1.03	0.10	1.28	0.21	1.03	0.24	0.97	0.03	IAP-like protein 1
AT1G71860.1	1.01	0.09	0.96	0.13	0.91	0.10	1.18	0.27	1.16	0.25	0.97	0.08	0.98	0.22	PTP1
AT3G02830.1	0.98	0.09	0.89	0.13	0.98	0.06	0.84	0.25	1.05	0.11	0.83	0.27	0.86	0.24	PENTA 1
AT4G39260.1	0.99	0.01	0.91	0.09	0.98	0.08	0.97	0.15	1.24	0.22	0.95	0.20	0.91	0.06	GRP8
AT5G57870.1	1.03	0.08	1.06	0.14	0.96	0.13	1.30	0.45	1.14	0.26	1.07	0.14	1.29	0.31	eIFiso4G1
AT2G37170.1	1.00	0.11	1.08	0.17	1.04	0.33	0.98	0.12	1.12	0.45	0.97	0.10	1.22	0.15	PIP2
AT4G35100.1	1.02	0.10	0.95	0.28	0.88	0.12	0.99	0.11	1.03	0.34	1.03	0.42	1.19	0.26	PIP3
AT1G21630.2	0.97	0.02	0.94	0.09	1.01	0.01	1.00	0.07	1.17	0.19	0.92	0.05	0.95	0.05	alcium-binding EF hand family protein
AT5G08050.1	0.89	0.10	0.86	0.08	0.84	0.18	1.16	0.25	1.11	0.19	1.09	0.16	1.17	0.14	Unknown
AT2G30930.1	0.92	0.02	0.90	0.17	0.88	0.14	0.91	0.07	1.09	0.09	0.75	0.17	0.87	0.05	Unknown
AT5G21940.1	1.20	0.16	1.54	0.68	1.51	0.20	0.89	0.09	1.39	0.34	1.01	0.26	1.47	0.38	Unknown
AT5G39570.1	0.96	0.13	1.00	0.30	0.91	0.14	0.76	0.35	1.24	0.51	2.22	2.70	1.54	1.24	Unknown
AT1G08680.4	1.05	0.08	1.05	0.10	0.98	0.09	0.91	0.06	1.13	0.05	0.99	0.22	1.06	0.19	AGD14
AT3G11330.1	0.98	0.05	1.05	0.36	1.13	0.28	1.20	0.40	1.04	0.24	1.00	0.14	1.12	0.32	it Intracellular Ras-group-related LRRs
AT1G28330.5	1.07	0.12	0.96	0.22	1.40	0.22	0.85	0.08	1.11	0.32	1.03	0.35	1.29	0.16	ormancy-associated protein (DRM1)
AT2G17980.1	0.97	0.15	1.10	0.09	0.99	0.15	1.00	0.12	1.03	0.14	1.05	0.08	1.34	0.18	SLY1 gene family
AT2G38410.1	1.00	0.14	1.02	0.24	1.05	0.22	1.25	0.34	0.97	0.09	1.00	0.19	1.22	0.19	ENTH/VHS/GAT family protein
AT4G24680.1	0.93	0.05	0.99	0.06	0.88	0.10	0.84	0.06	0.97	0.09	1.46	1.18	0.84	0.19	Modifier of SNC1
AT4G27450.1	1.07	0.09	0.93	0.18	1.32	0.28	1.02	0.13	1.09	0.39	1.06	0.14	1.31	0.22	n induced protein with YGL and LRDR motifs
AT5G08610.1	1.06	0.01	1.05	0.17	1.06	0.21	1.16	0.10	1.04	0.05	2.60	2.45	1.09	0.05	ucleoside triphosphate hydrolases superfamily protein
AT5G09620.1	0.98	0.10	0.94	0.15	0.94	0.15	0.87	0.24	1.08	0.18	0.87	0.30	0.91	0.25	cosapeptide/Phox/Bem1p family protein
AT5G59210.1	0.94	0.02	0.83	0.15	0.81	0.07	0.88	0.02	0.97	0.19	0.86	0.08	0.86	0.01	myosin heavy chain-related
AT5G67220.1	1.07	0.09	1.20	0.10	1.17	0.15	1.24	0.19	1.09	0.12	1.23	0.29	1.25	0.04	nked oxidoreductases superfamily protein
AT3G15450.1	1.27	0.28	1.15	0.31	1.69	0.46	0.98	0.07	1.38	0.27	1.20	0.32	1.65	0.41	n induced protein with YGL and LRDR motifs
AT5G35430.1	0.90	0.35	1.11	0.64	0.91	0.52	0.84	0.23	0.86	0.02	1.19	0.51	1.44	0.95	peptide repeat (TPR)-like superfamily protein
AT2G43790.1	1.12	0.01	1.08	0.10	0.91	0.20	1.03	0.07	1.17	0.10	1.24	0.15	1.45	0.53	MAPK6
**Phosphorylation Level down regulated in Col-0**															
AT2G27710.1	1.06	0.04	0.97	0.13	1.06	0.11	1.08	0.11	1.14	0.35	0.97	0.07	1.02	0.06	60S acidic ribosomal protein family
AT3G09200.1	0.98	0.11	1.00	0.25	1.07	0.14	1.08	0.09	1.11	0.38	1.05	0.06	1.09	0.03	Ribosomal protein L10 family protein
AT1G05805.1	1.06	0.44	0.95	0.21	1.00	0.22	0.78	0.43	0.87	0.38	0.81	0.58	0.78	0.50	AKS2
AT5G17170.1	0.97	0.06	0.95	0.19	0.94	0.09	0.85	0.17	1.00	0.11	0.99	0.35	1.09	0.41	SOS3-1
AT5G13850.1	0.95	0.07	0.88	0.13	1.03	0.23	0.85	0.30	1.29	0.32	0.90	0.33	0.81	0.20	NACA3
AT5G55210.1	1.08	0.16	1.03	0.22	1.02	0.12	0.84	0.12	1.27	0.31	0.91	0.30	1.07	0.38	Unknown
AT1G22530.1	1.02	0.08	1.05	0.14	0.88	0.21	0.96	0.02	1.00	0.23	0.95	0.02	1.32	0.53	Unknown
AT1G70570.2	1.01	0.08	0.98	0.15	1.00	0.09	1.05	0.36	1.13	0.22	1.08	0.04	1.03	0.11	nthranilate phosphoribosyltransferase
AT3G23490.1	1.04	0.06	0.93	0.19	1.02	0.14	1.17	0.24	1.24	0.24	1.03	0.14	1.06	0.27	Cyanase

Supplemental Table 7. Phosphorylation motifs identified in whole seeding of *Arabidopsis* under submergence.

Group	Motif	Motif Score	Submergence regulated	Putative kinase
I				GSK3
	.....GSP.....	26.24		
	......SP.....	16	yes	
	......S.S....	11.42		
	...S..S......	5.49		
	......S.G....	5.67		
II				CDK5
	......SSP....	16.53		
	......S...SP.	14.8	yes	
III				PKA
	...KS.S......	14.26		
	...RS.S......	17.24		

Supplemental Table 8. Primer lists

Gene Name	AGI number	Forward primer (5'to3')	Reverse primer (5'to3')
**Primers for Q-PCR**			
SnRK1.1	At3g01090	CTTGACAACATTGGGCGACTTA	CTTTGGAGACGAGTCCAGCATAA
SnRK1.2	At3g29160	AGTGCAGCTGATTGTTGACCAT	AATGTCGATGGGTTCCTGGT
ZAT10	At1g27730	CTGTTGAGAGTTGTGTAGGAATTTGTTG	CAAGAAGAATTGGAATCAGTCAAAGTC
HSFA2	At2g26150	GGAAGCAGCGTTGGATGTG	GGCTGTCCCAATCCAAAGG
TUB3	AT5G62700	GGTTGGTTTTGCTCCTCTCACC	TAGCGTCCGTGCCTTGGGTC
ADH1	At1g77120	CATGAACAAGGAGCTGGAGCTTG	CTCTCCCTTCAGCATGTAATCAAAGG
**Primers for RT-PCR**			
SnRK1.1	At3g01090	CATGGAAGGTACTCCCCGTA	GCAGCACACAGATCCAAGAA
SnRK1.2	At3g29160	TCACAAGTCCCTGTTGATCG	ACCCGGCAAAGTAAACACTG
akin beta1	At5g2117	TGATAATCCCCCAGAGCAAG	CACATGTTGAGGCTTTGTGG
akin beta2	At4g16360	GATTGCTGTGGAGGGATCAT	AATCGGTGAGTGGAACCAAG
akin beta3	At3g48530	TGTTTTCGAAGGACAGCAAA	GTTGAAGGTGTGGCGGTACT
akin R	At3g48530	CATCCCGGTGGTTGATTTAG	GTTCTTCGTGCAAGCAATCA
akin beta R	At4g16360	TTGAGACCTCACGCCTCTTT	GGTTGTTTTGCTGTGGGAGT
β-ATP	At5g08680	GATCATGACATCTCTCGAGG	TGGTAAGGAGCAAGGAGATC
**Primers for Cloning**			
F2KP	At1g07110	ATGGGGTCAGGTGCATC	GTCCATGAGT TTGTAGCGTT TC
eIFiso4G1	At5g57870	ATGCAGCAAGGCGATC	CAGATTCCGACAAGCC
PTP1	At1g71860	ATGGCGACCGGTAAAAC	GGAACTCGTTCCAGCATTTG
PENTA	At3g02830	ATGGATTTTAATGCCGGAGTT	CTGCTGT GTATTATCAA TGGTAGT
**Primers for site mutagenesis**			
SnRK1.1T175A	At3g01090	GCGAGATGGTCATTTTTTGAAGGCAAGTTGTGGAAGTCCA	TGGACTTCCACAACTTGCCTTCAAAAAATGACCATCTCGC
SnRK1.1T175D	At3g01090	GCGAGATGGTCATTTTTTGAAGGACAGTTGTGGAAGTCCA	TGGACTTCCACAACTGTCCTTCAAAAAATGACCATCTCGC
SnRK1.1K48M	At3g01090	ACATAAGGTTGCTATCATGATCCTCAATCGTCGCAAA	TTTGCGACGA TTGAGGATCA TGATAGCAAC CTTATGT
PTP1S7AS8A	At1g71860	GCGACCGGTAAAACCGCTGCCGCCGCGAATCTTTTCACT	AGTGAAAAGA TTCGCGGCGG CAGCGGTTTT ACCGGTCGC
PTP1S7DS8D	At1g71860	GCGACCGGTAAAACCGACGACGCCGCGAATCTTTTCACT	AGTGAAAAGA TTCGCGGCGT CGTCGGTTTT ACCGGTCGC
